# Supplementary material for: Tumor-targeted glutathione oxidation catalysis with ruthenium nanoreactors against hypoxic osteosarcoma
Source: Nat Commun. 2024 Oct 31;15:9405. doi: 10.1038/s41467-024-53646-y (PMC11526146; doi:10.1038/s41467-024-53646-y)
Supplement: Supplementary file 1 — Supplementary Information [file 41467_2024_53646_MOESM1_ESM.pdf]

Supplementary Information

Tumor-Targeted Glutathione Oxidation Catalysis with Ruthenium Nanoreactors against Hypoxic Osteosarcoma

Hanchen Zhang[1,2], Nicolás Montesdeoca[3], Dongsheng Tang[1,2], Ganghao Liang[1,2] Minhui Cui[1,2], Chun Xu[4], Lisa-Marie Servos[3], Tiejun Bing,[5] Zisis Papadopoulos[3], Meifang Shen,[6] Haihua Xiao* [1,2], Yingjie Yu*[6] and Johannes Karges*[3]

[1] Beijing National Laboratory for Molecular Sciences, Laboratory of Polymer Physics and Chemistry, Institute of Chemistry, Chinese Academy of Sciences, Beijing, 100190, China

[2] University of Chinese Academy of Sciences, Beijing 100049, China

[3] Faculty of Chemistry and Biochemistry, Ruhr-University Bochum, Universitätsstrasse 150, 44780 Bochum, Germany

[4] School of Dentistry, The University of Queensland, Brisbane, Queensland, Australia

[5] Immunology and Oncology center, ICE Bioscience, Beijing 100176, China

[6] State Key Laboratory of Organic-Inorganic Composites, Beijing Laboratory of Biomedical Materials, Beijing University of Chemical Technology, Beijing, 100029, China

Table of Contents

Supplementary Figure 1 1

Supplementary Figure 2 2

Supplementary Figure 3 3

Supplementary Figure 4 4

Supplementary Figure 5 5

Supplementary Figure 6 6

Supplementary Figure 7. 7

Supplementary Figure 8 8

Supplementary Figure 9 9

Supplementary Figure 10 10

Supplementary Figure 11 11

Supplementary Figure 12 12

Supplementary Figure 13 13

Supplementary Figure 14 14

Supplementary Figure 15 15

Supplementary Figure 16 16

Supplementary Figure 17 17

Supplementary Figure 18 18

Supplementary Figure 19 10

Supplementary Figure 20 20

Supplementary Figure 21 21

Supplementary Figure 22 22

Supplementary Figure 23 23

Supplementary Figure 24 24

Supplementary Figure 25 25

Supplementary Figure 26 26

Supplementary Figure 27 27

Supplementary Figure 28 28
Supplementary Figure 29 29
Supplementary Figure 30 30
Supplementary Figure 31 31
Supplementary Figure 32 32
Supplementary Figure 33 33
Supplementary Figure 34 34
Supplementary Figure 35 35
Supplementary Figure 36 36
Supplementary Figure 37 37
Supplementary Figure 38 38
Supplementary Figure 39 39
Supplementary Figure 40 40
Supplementary Figure 41 41
Supplementary Figure 42 42
Supplementary Table 1.. 43
Supplementary Table 2.. 44
Supplementary Table 3.. 45
Supplementary Table 4.. 47
Supplementary Table 5.. 50
Supplementary Table 6.. 54

Supplementary Figures and Tables

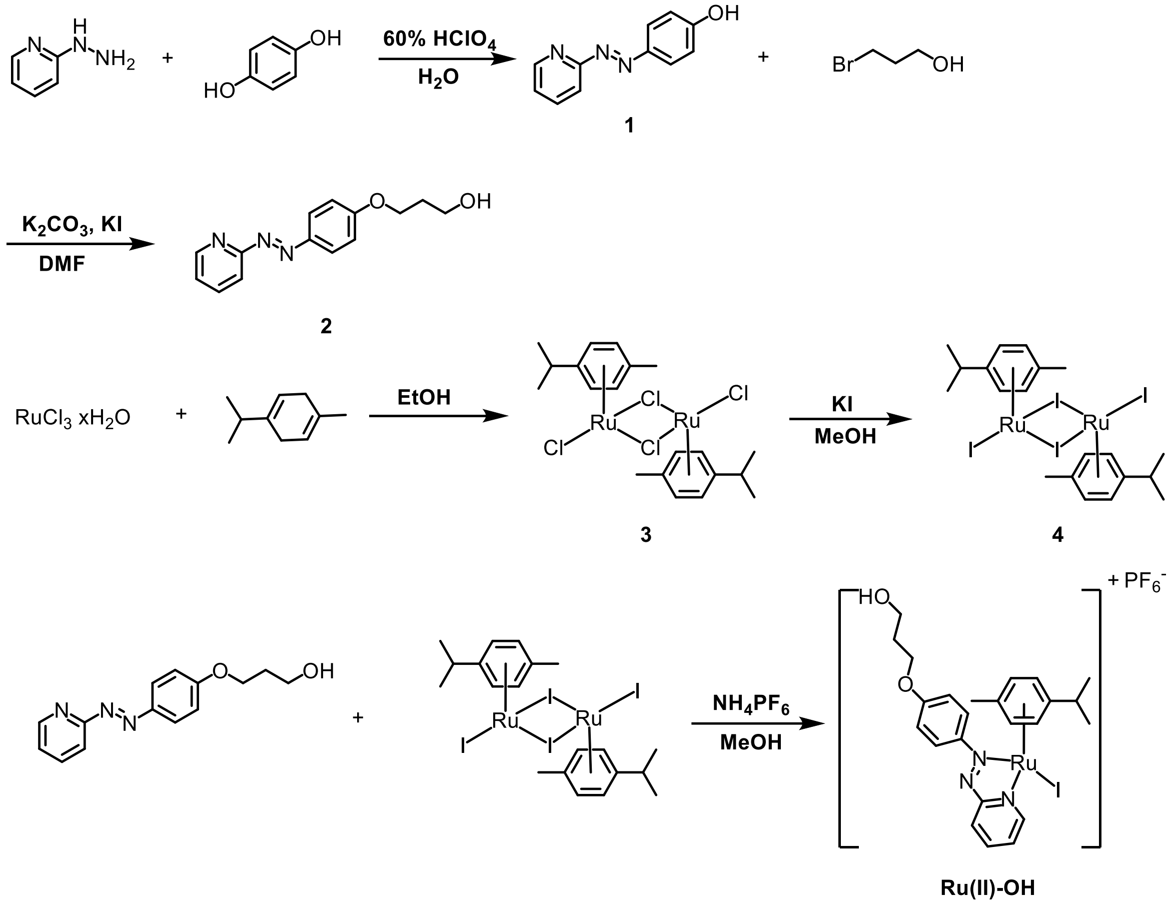

Supplementary Figure 1. Synthetic route for the ruthenium (Ru) arene complex (Ru(II)-OH).

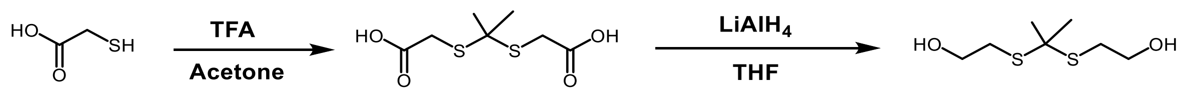

Supplementary Figure 2. Synthetic route for 2,2'-(propane-2,2 diylbis(sulfanediyl))bis(ethan-1-ol).

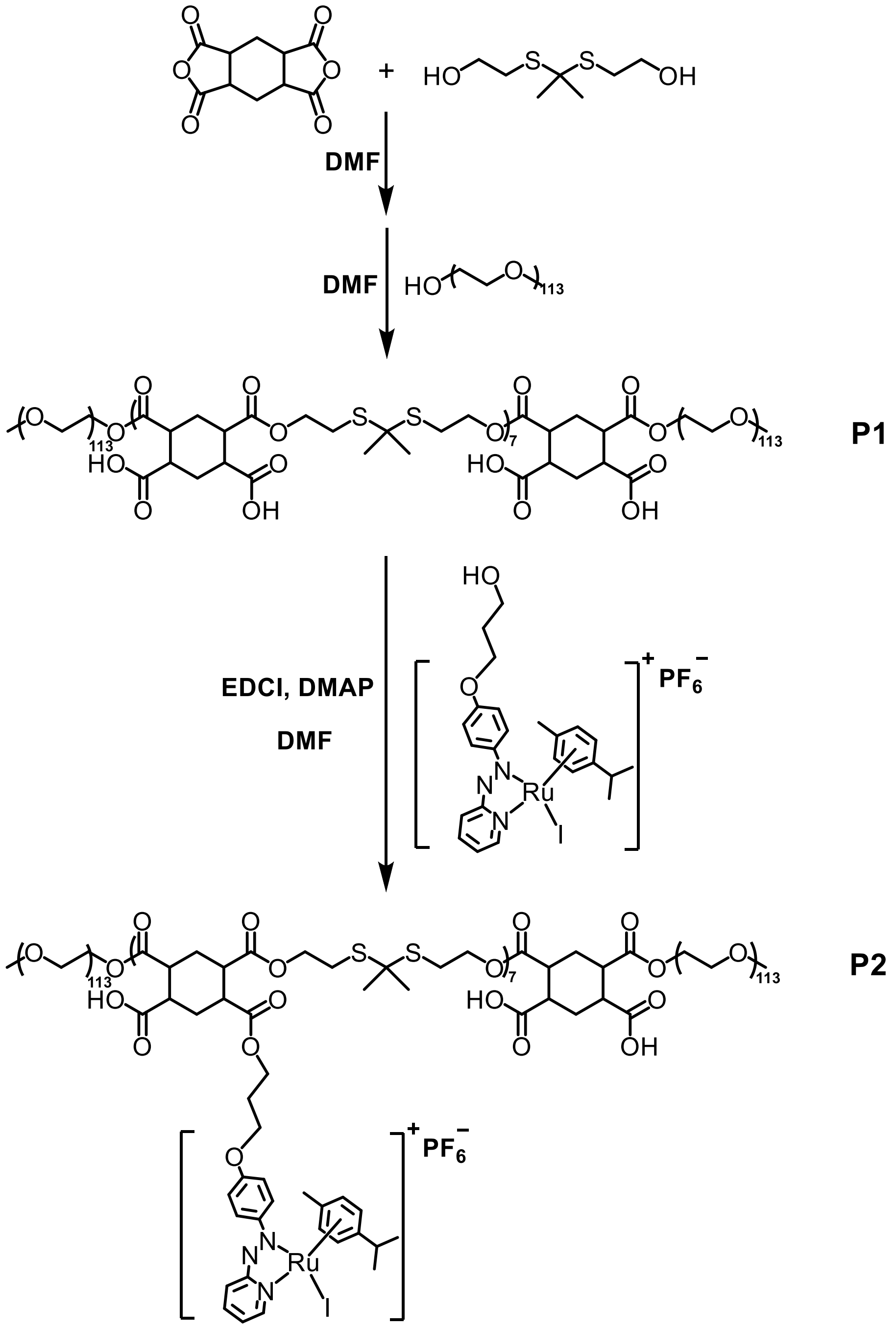

Supplementary Figure 3. Synthetic route for P1 and P2.

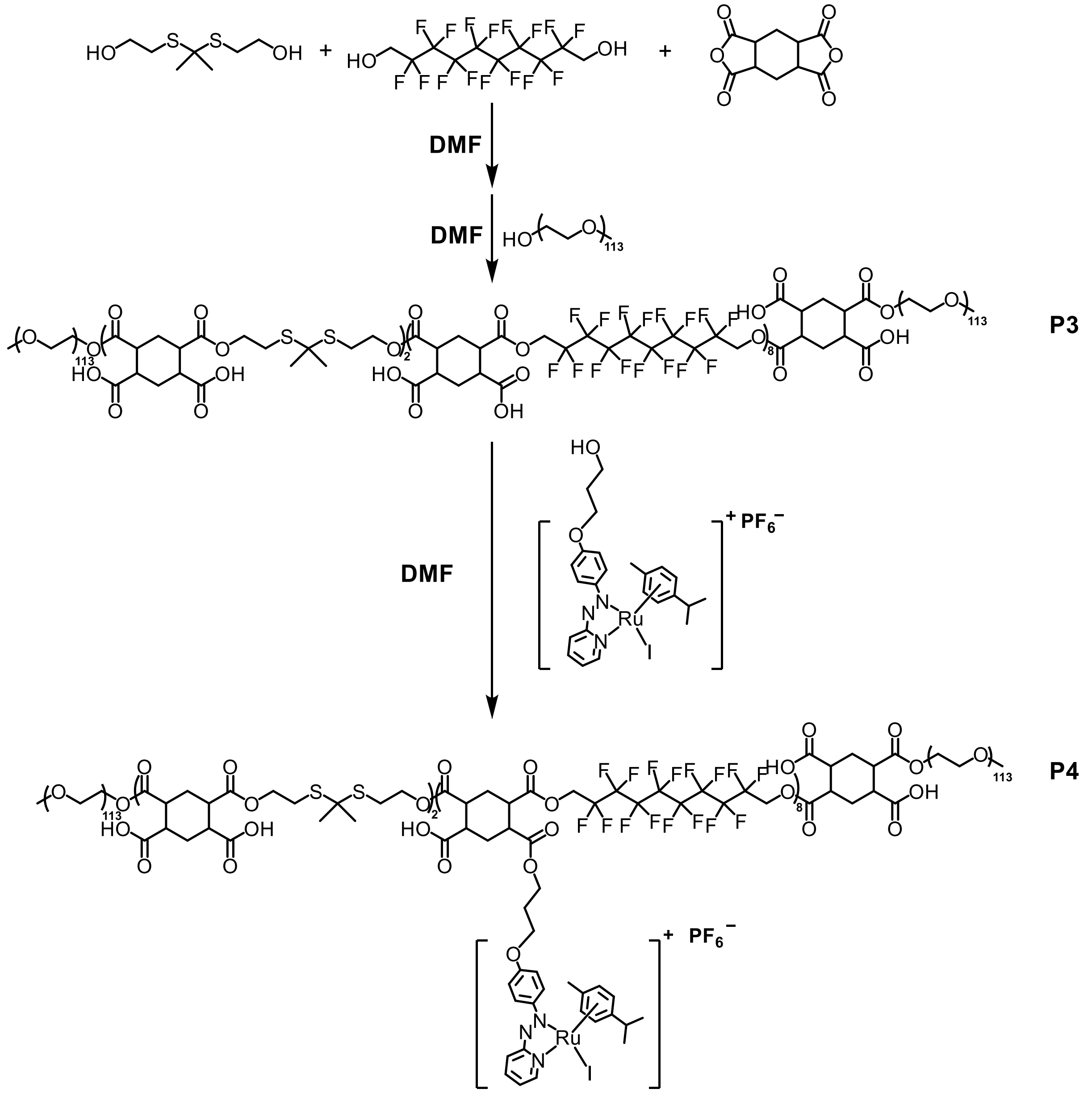

Supplementary Figure 4. Synthetic route for P3 and P4.

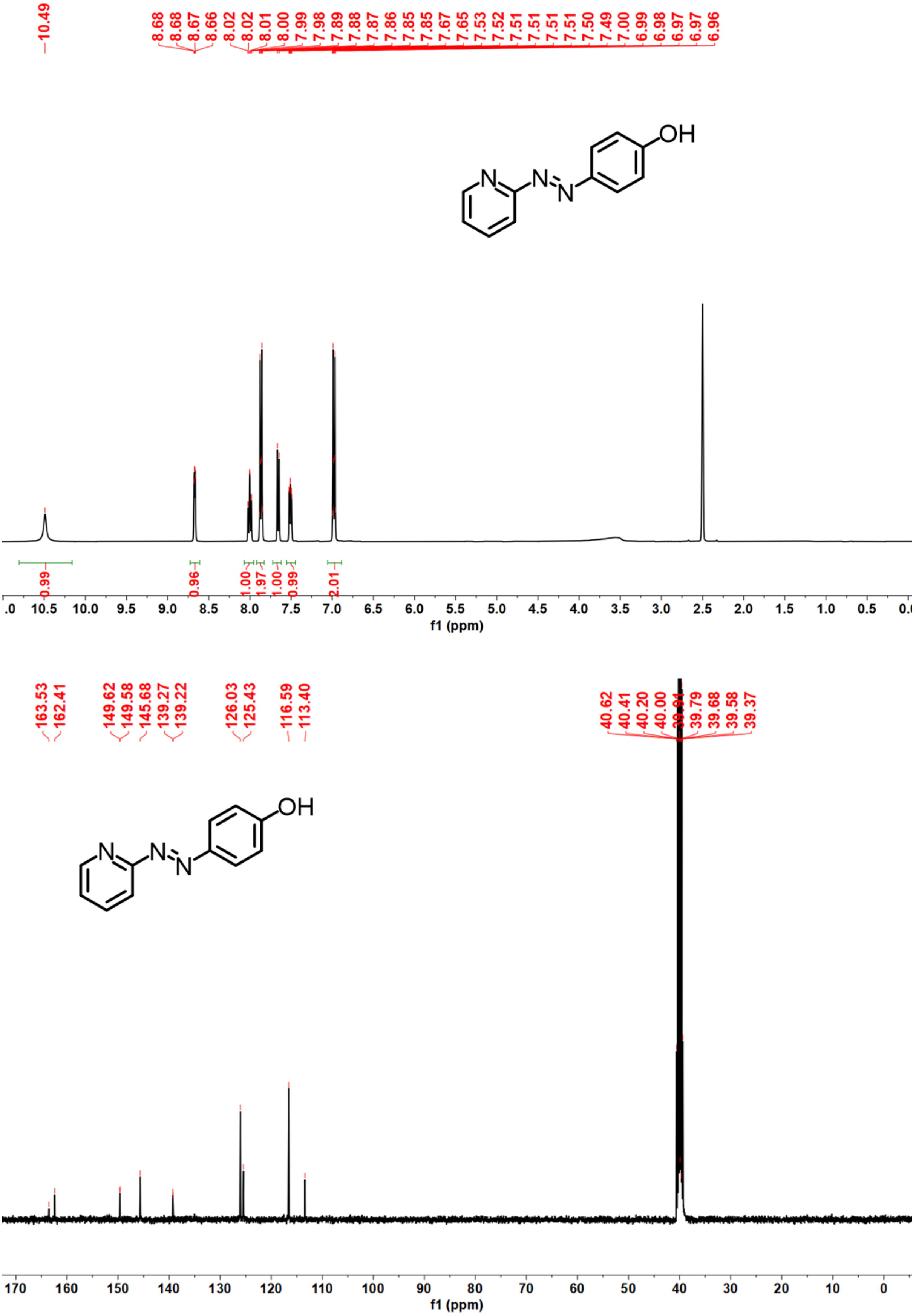

Supplementary Figure 5. ^{1}H-NMR and ^{13}C-NMR spectrums of 4-(pyridin-2-yldiazenyl)phenol in DMSO-d_6.

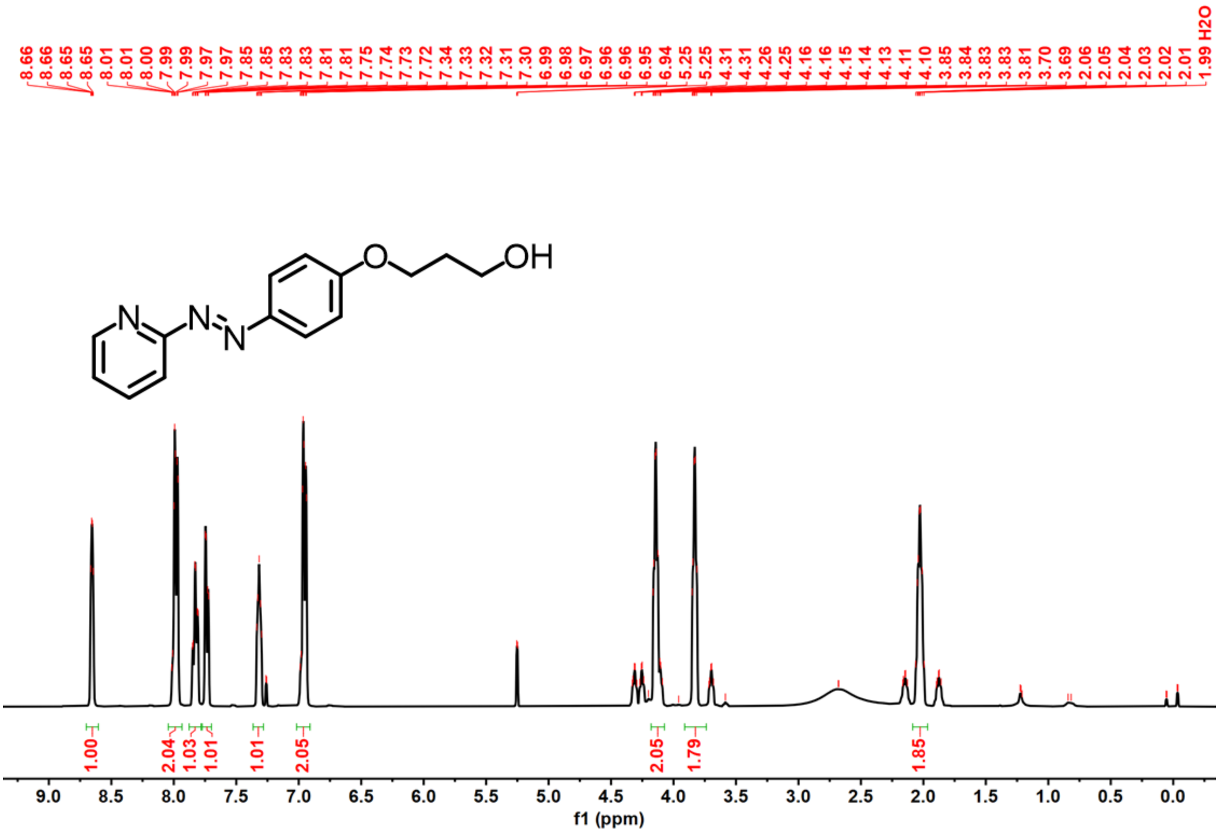

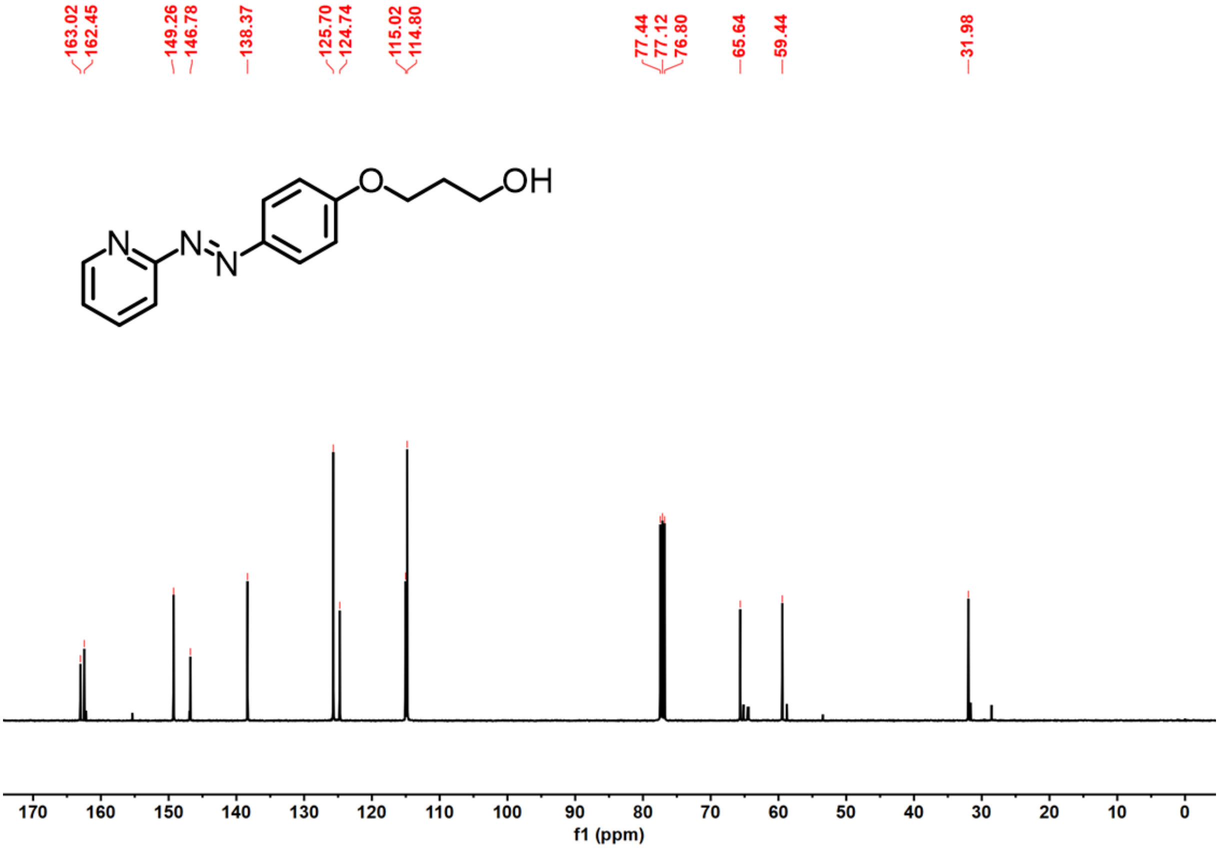

Supplementary Figure 6. ^{1}H-NMR and ^{13}C-NMR spectrums of 3-(4-(pyridin-2-yldiazenyl)phenoxy)propan-1-ol in MeOH-d_4.

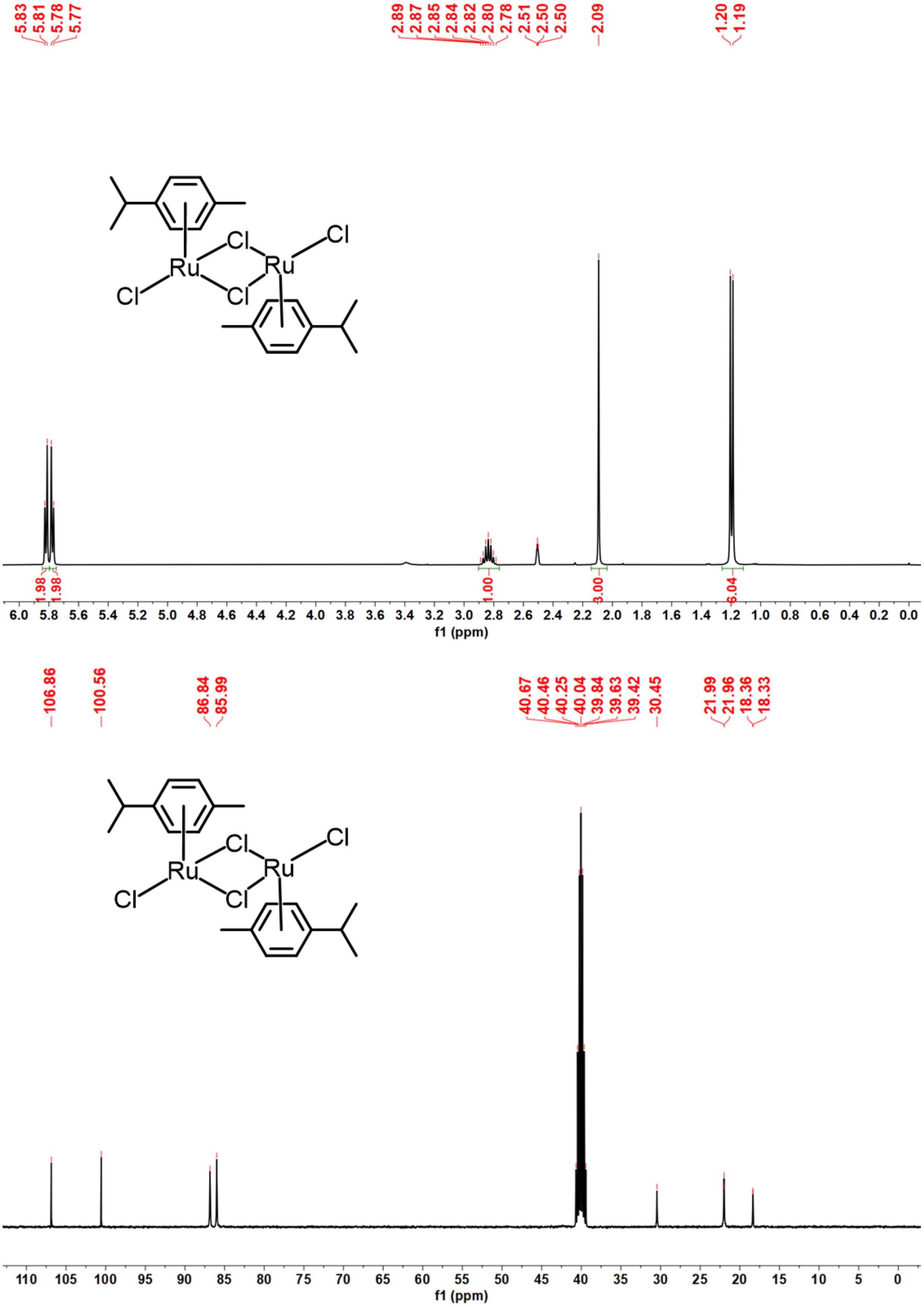

Supplementary Figure 7. ^{1}H-NMR and ^{13}C-NMR spectrums of dichloro(p-cymene)ruthenium(II) dimer in DMSO-d_6.

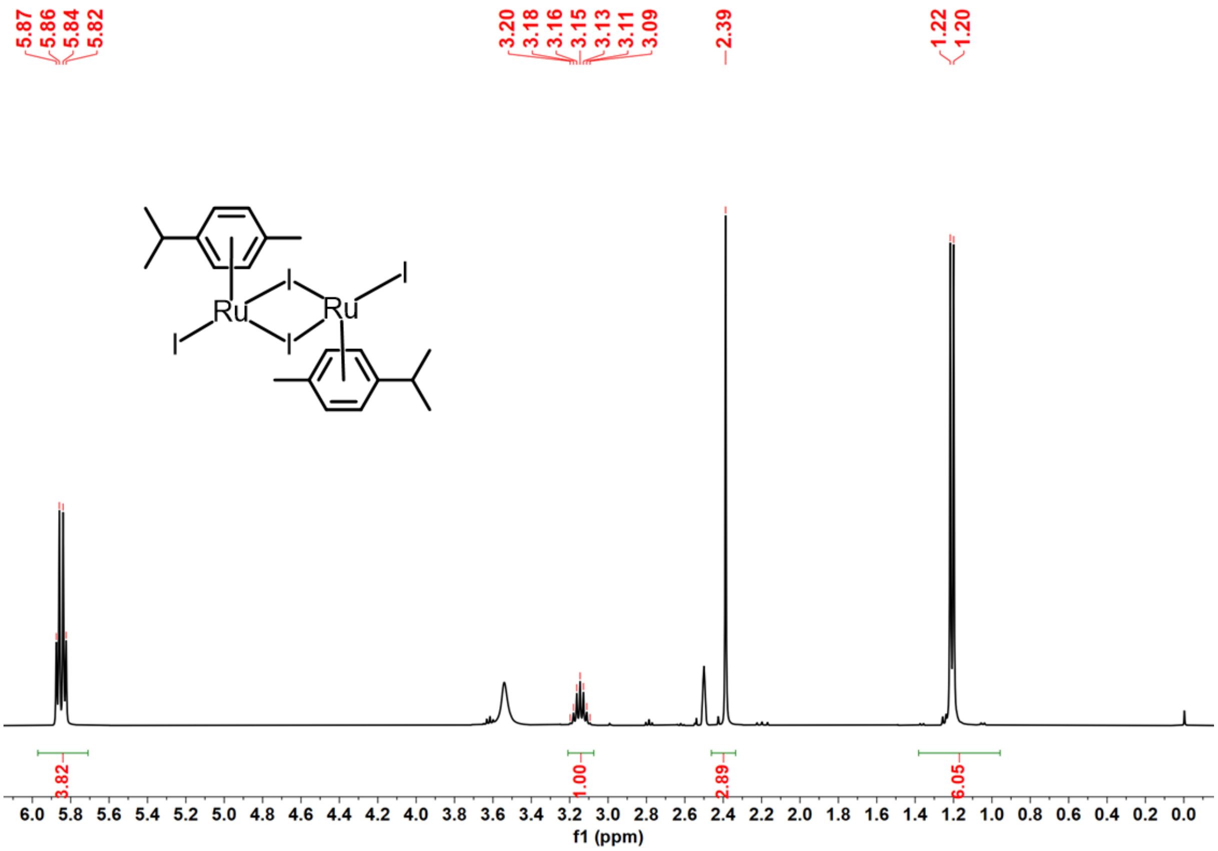

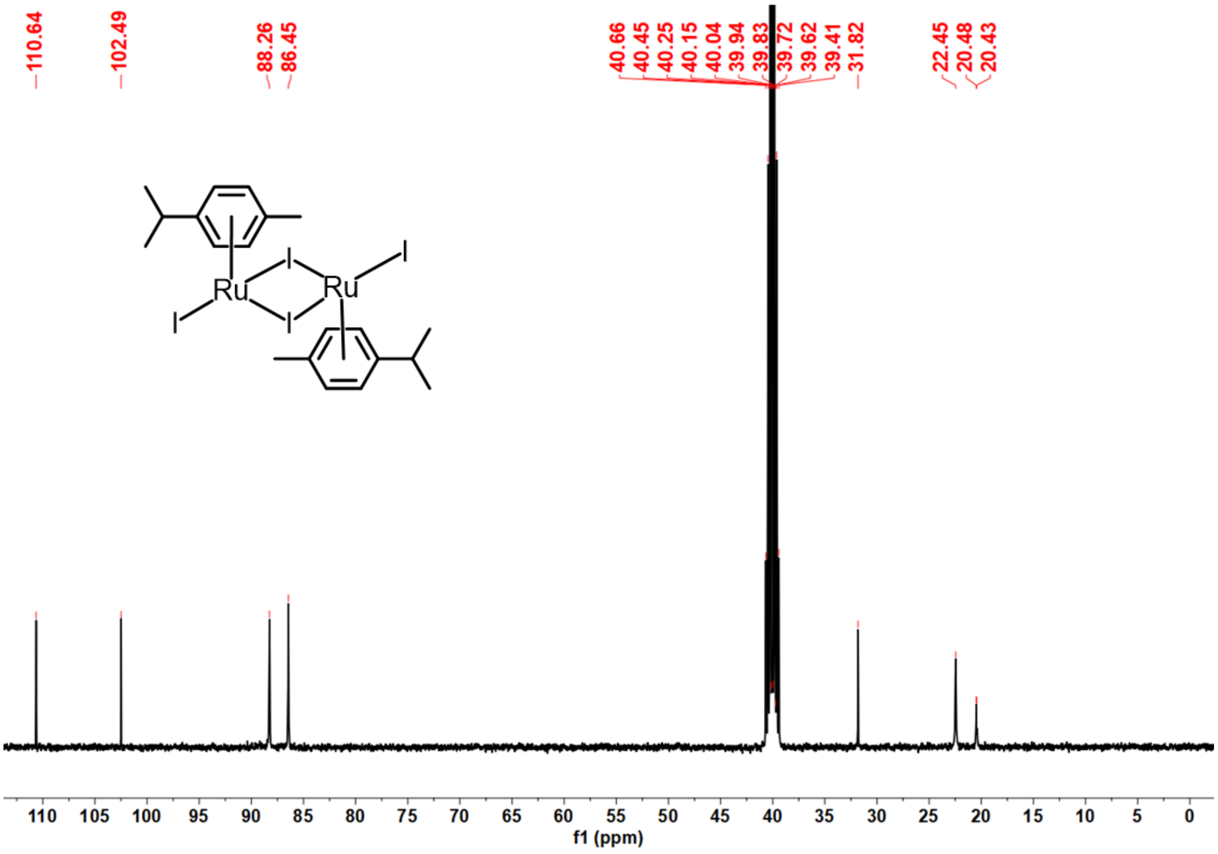

Supplementary Figure 8. ^{1}H-NMR and ^{13}C-NMR spectrums of diiodo(p-cymene)ruthenium(II) dimer in DMSO-d$_6$.

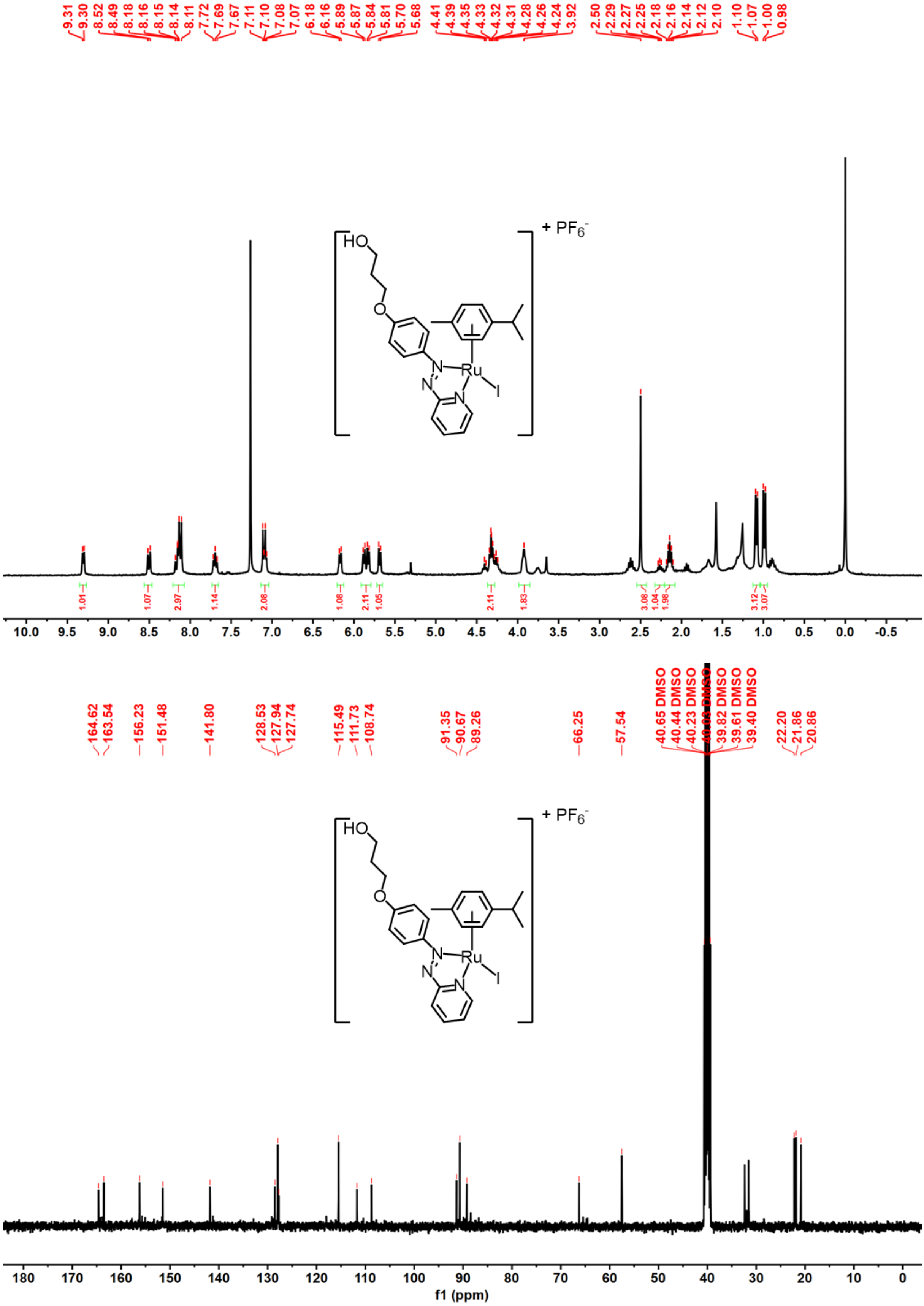

Supplementary Figure 9. ^{1}H-NMR and ^{13}C-NMR spectrums of Ru(II)-OH in CD_3Cl-d_3.

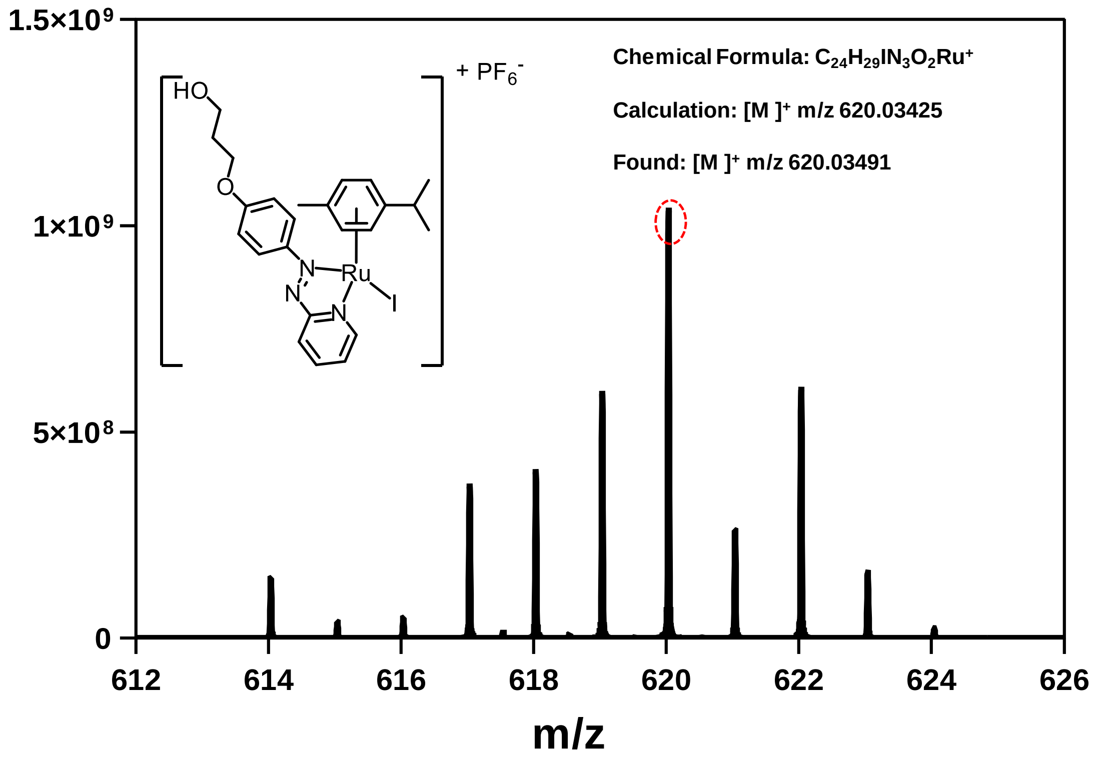

Supplementary Figure 10. High-resolution mass spectrum (HR-MS) of Ru(II)-OH.

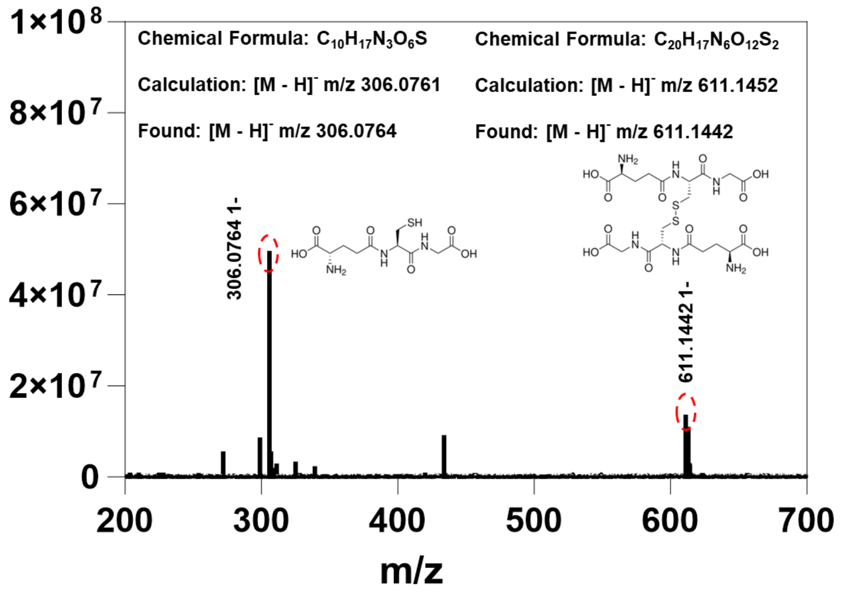

Supplementary Figure 11. HR-MS of the mixture of Ru(II)-OH and glutathione (GSH).

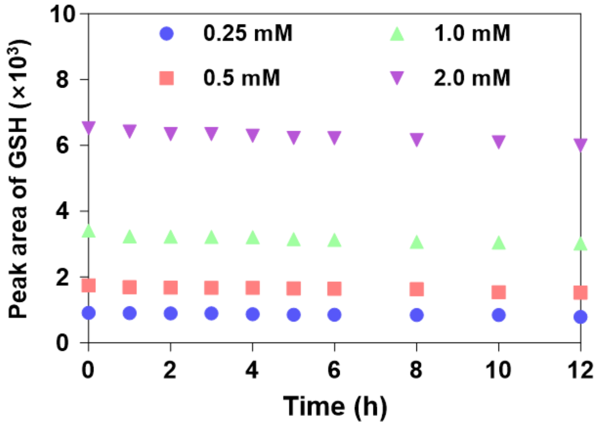

Supplementary Figure 12. Monitoring of possible changes in the GSH concentration upon incubation of a solution of GSH in phosphate-buffered saline (PBS) without the catalyst.

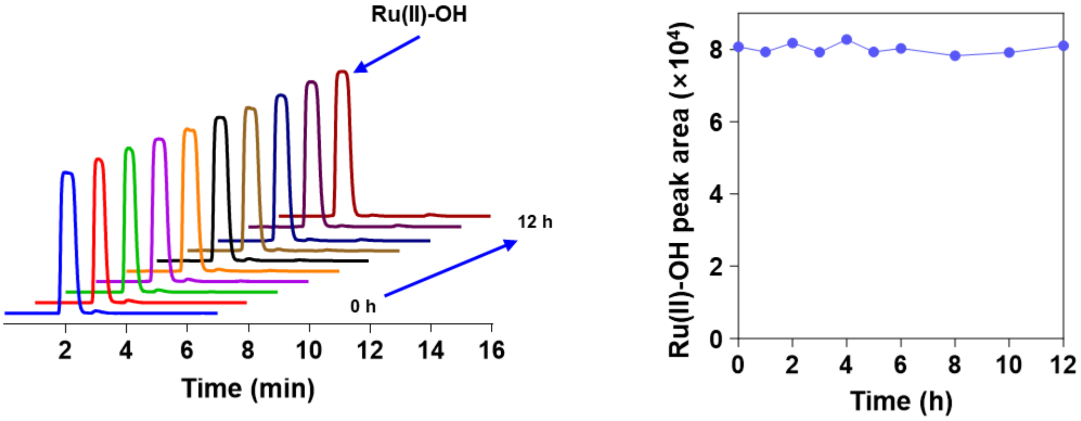

Supplementary Figure 13. Monitoring of the concentration of Ru(II)-OH during the catalytic transformation.

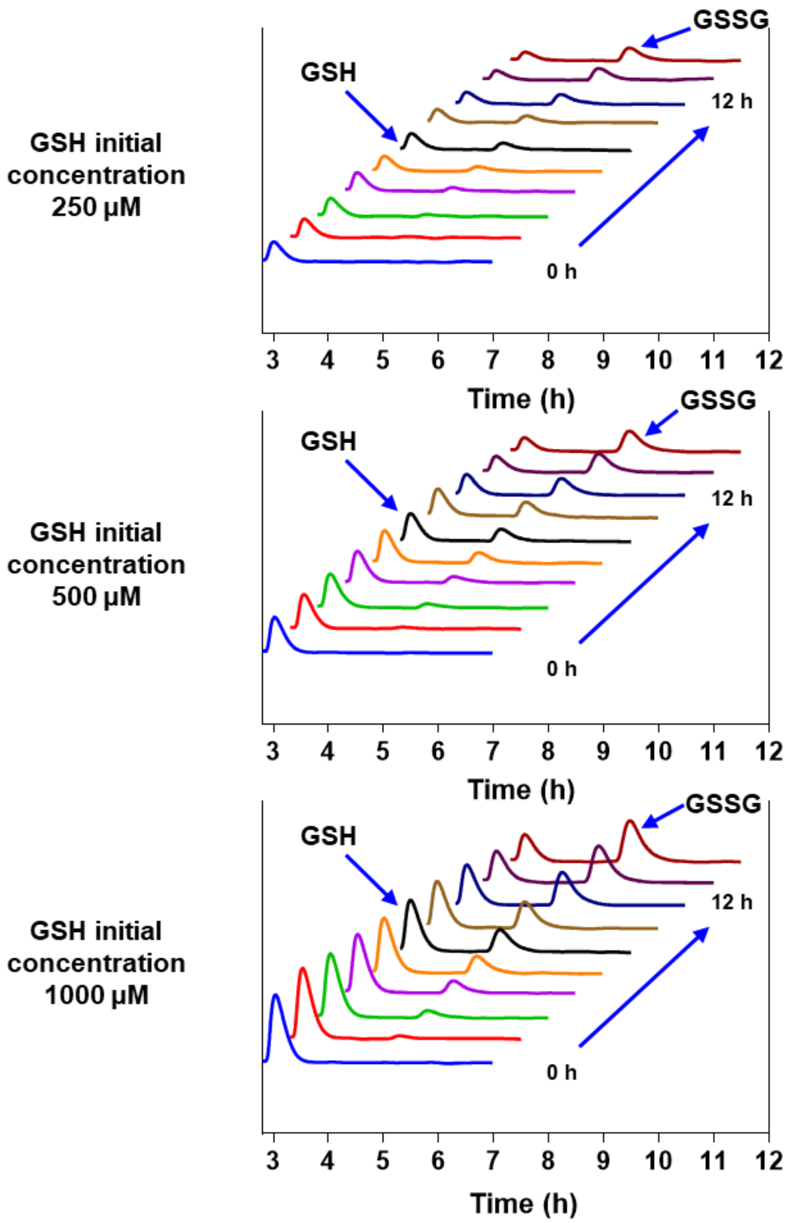

Supplementary Figure 14. Ultra performance liquid chromatography (UPLC) chromatograms of the conversion of GSH (250 µM, 500 µM, 1000 µM) to glutathione disulfide (GSSG) in the presence of Ru(II)-OH (50 µM).

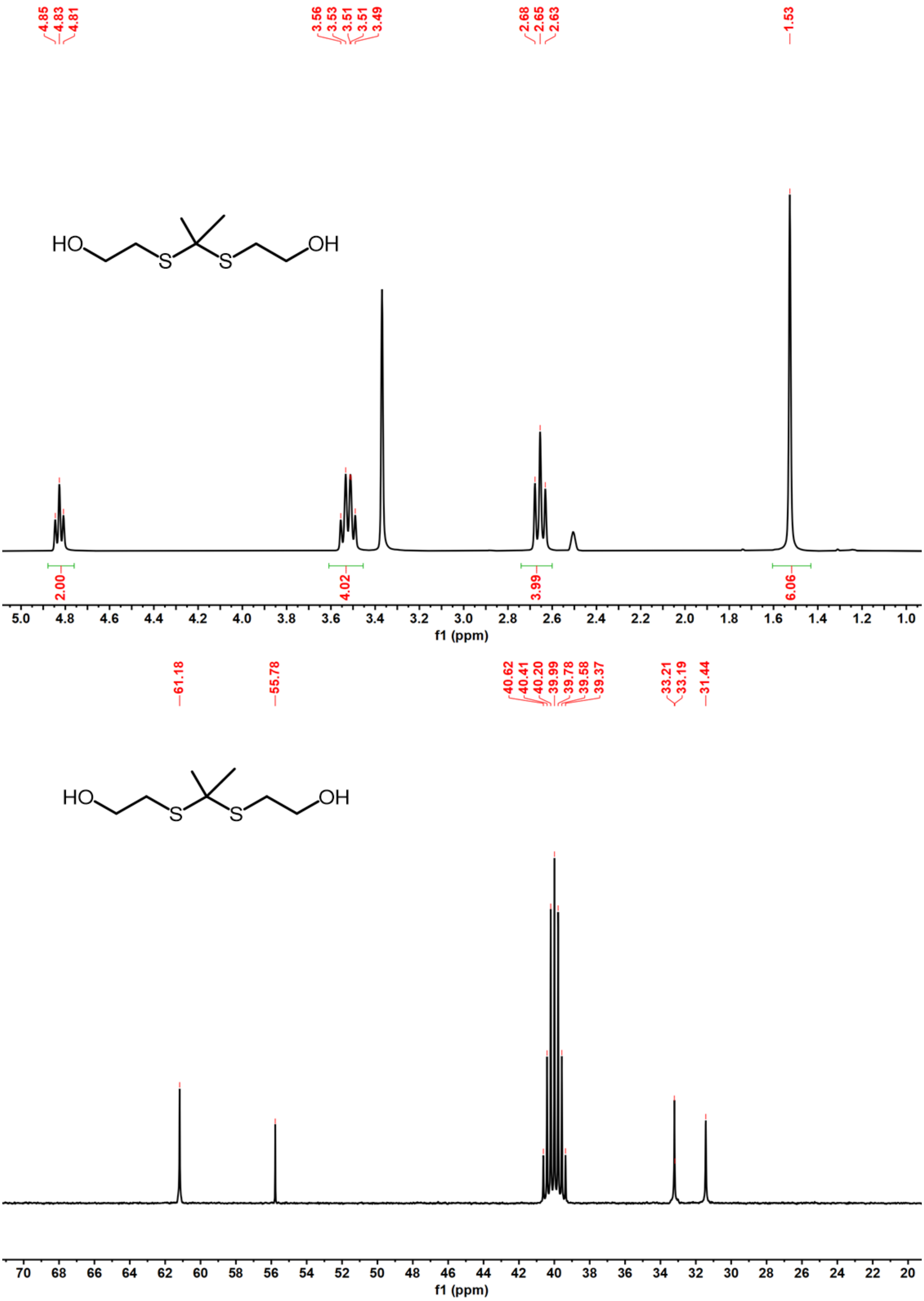

Supplementary Figure 15. ^{1}H-NMR and ^{13}C-NMR spectrums of 2,2'-(propane-2,2-diylbis(sulfanediyl))bis(ethan-1-ol) in DMSO-d_6.

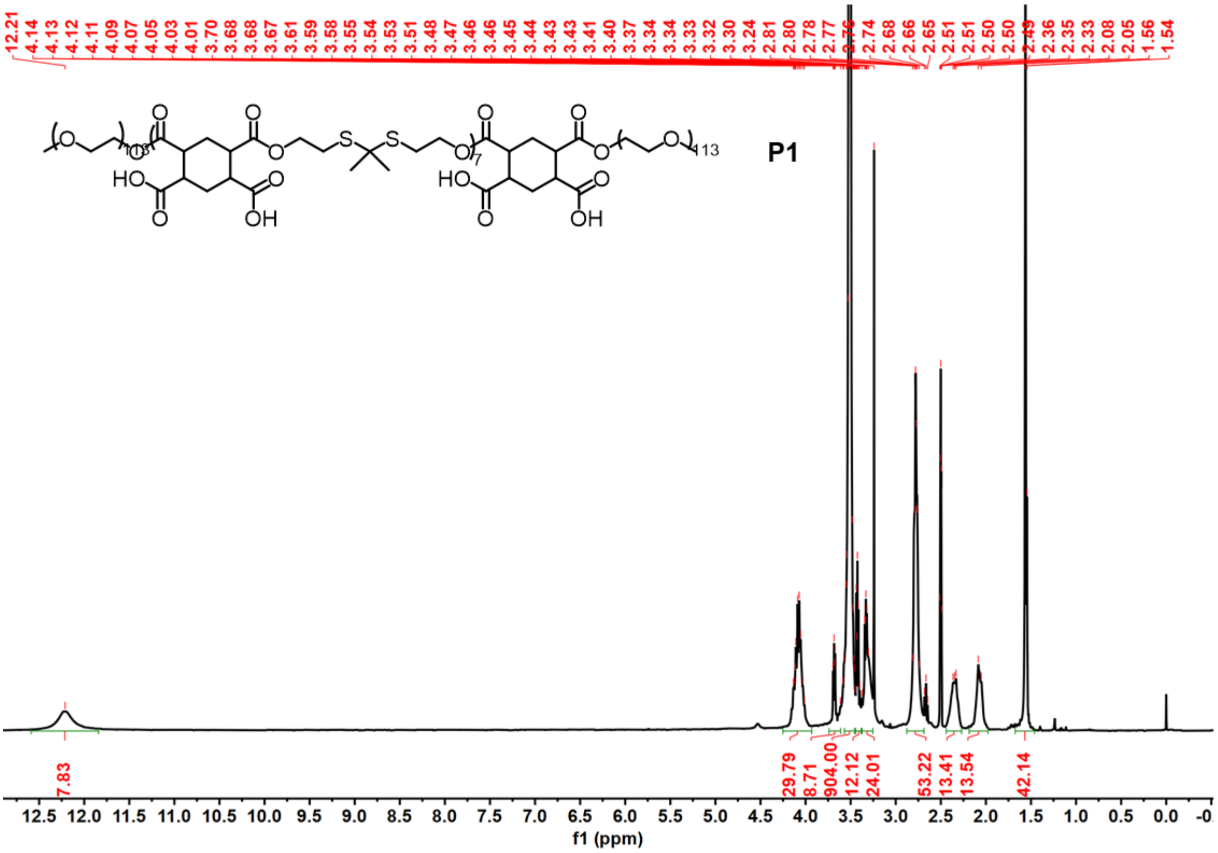

Supplementary Figure 16. ^{1}H-NMR spectrum of P1 in DMSO-d_6.

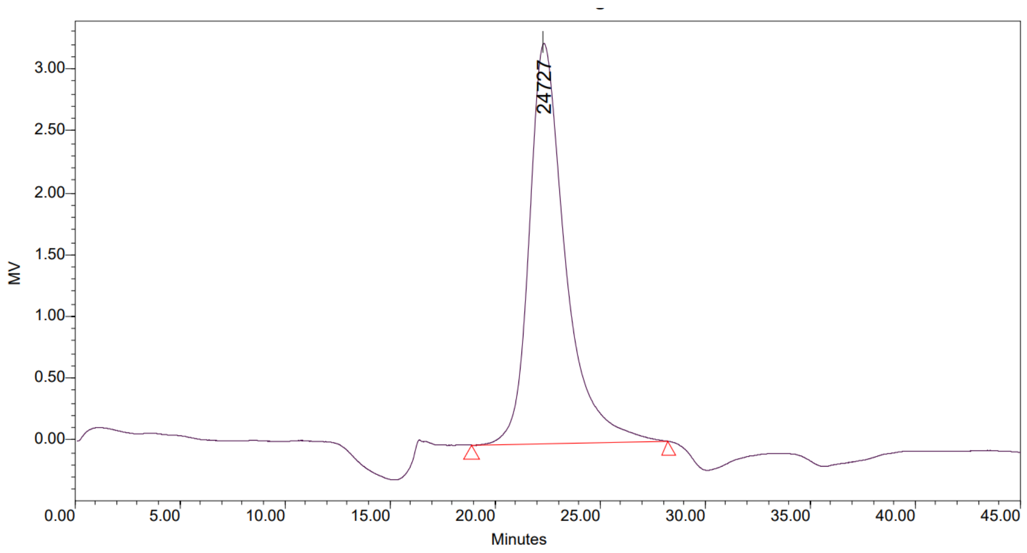

Supplementary Figure 17. Gel permeation chromatogram (GPC) of P1 in N, N-dimethylformamide (DMF).

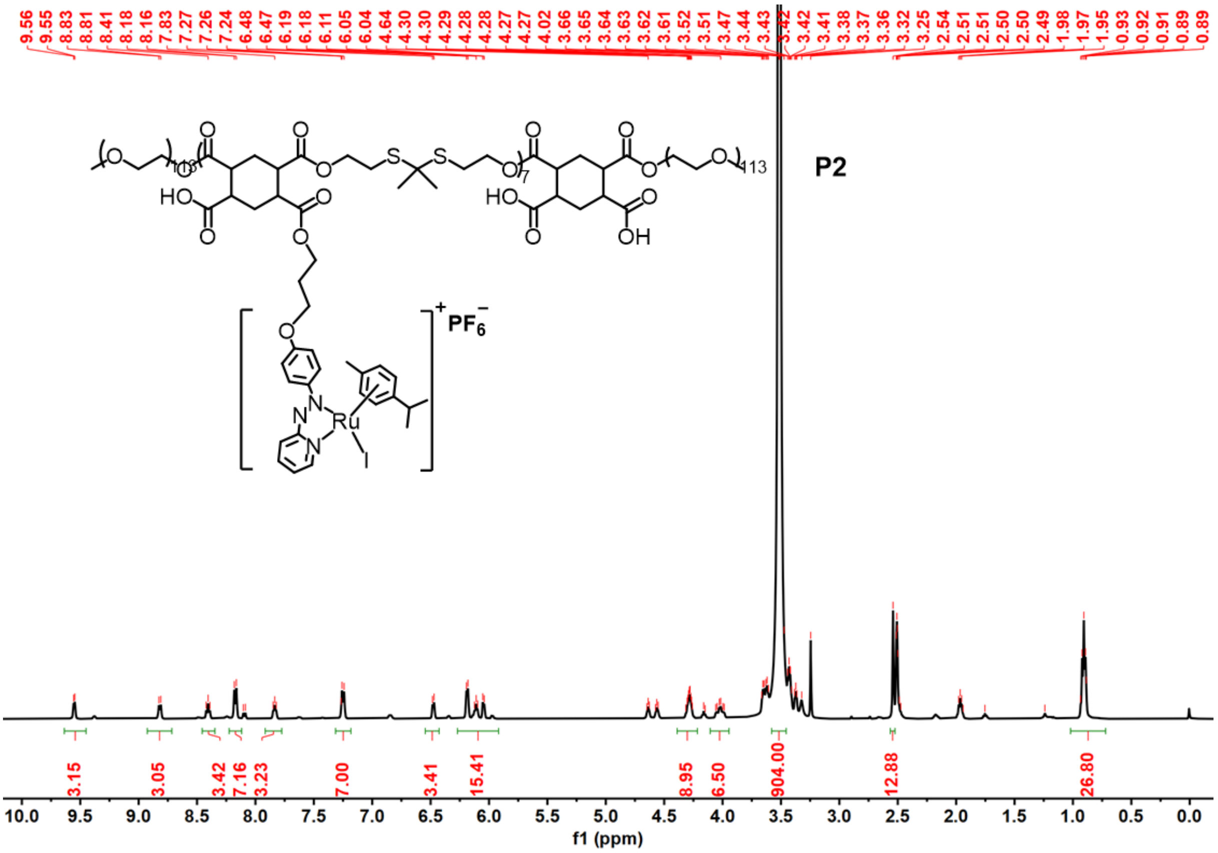

Supplementary Figure 18. ^{1}H-NMR spectrum of P2 in DMSO-d_6.

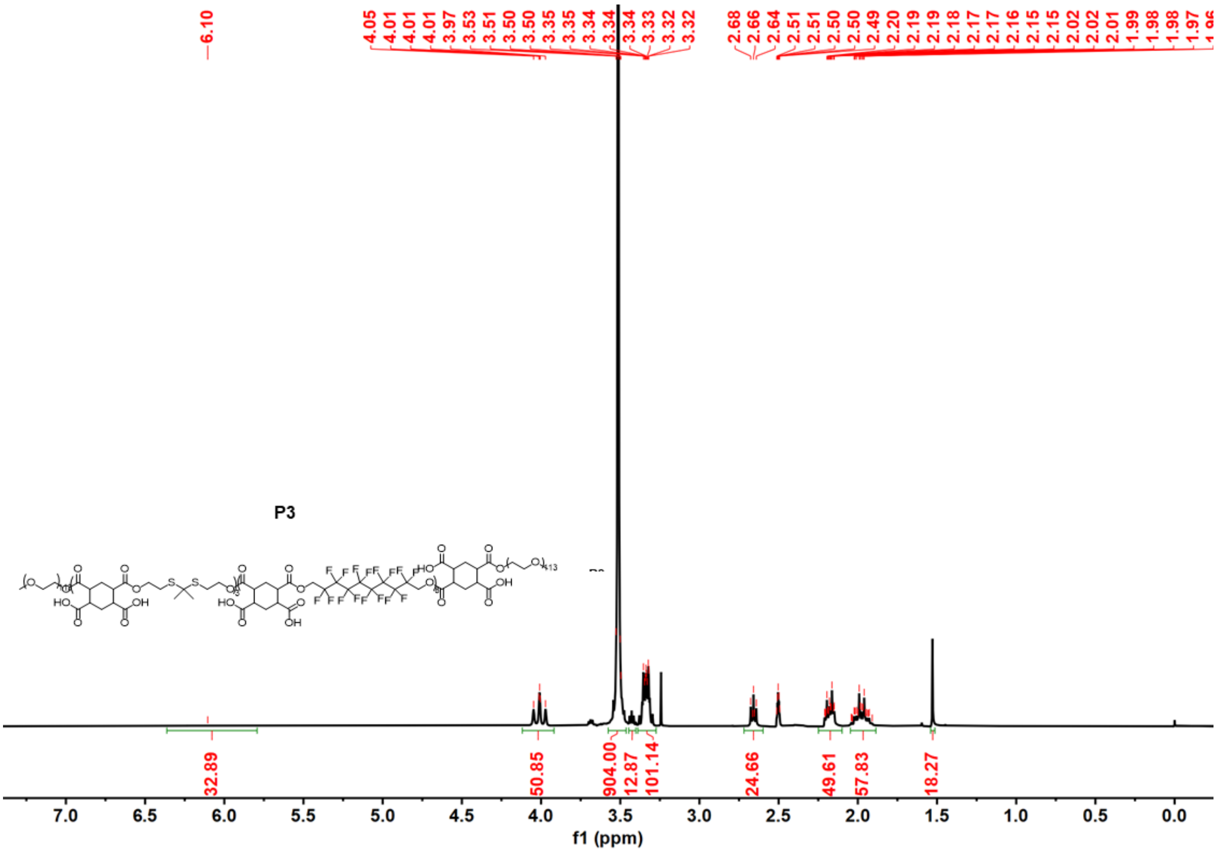

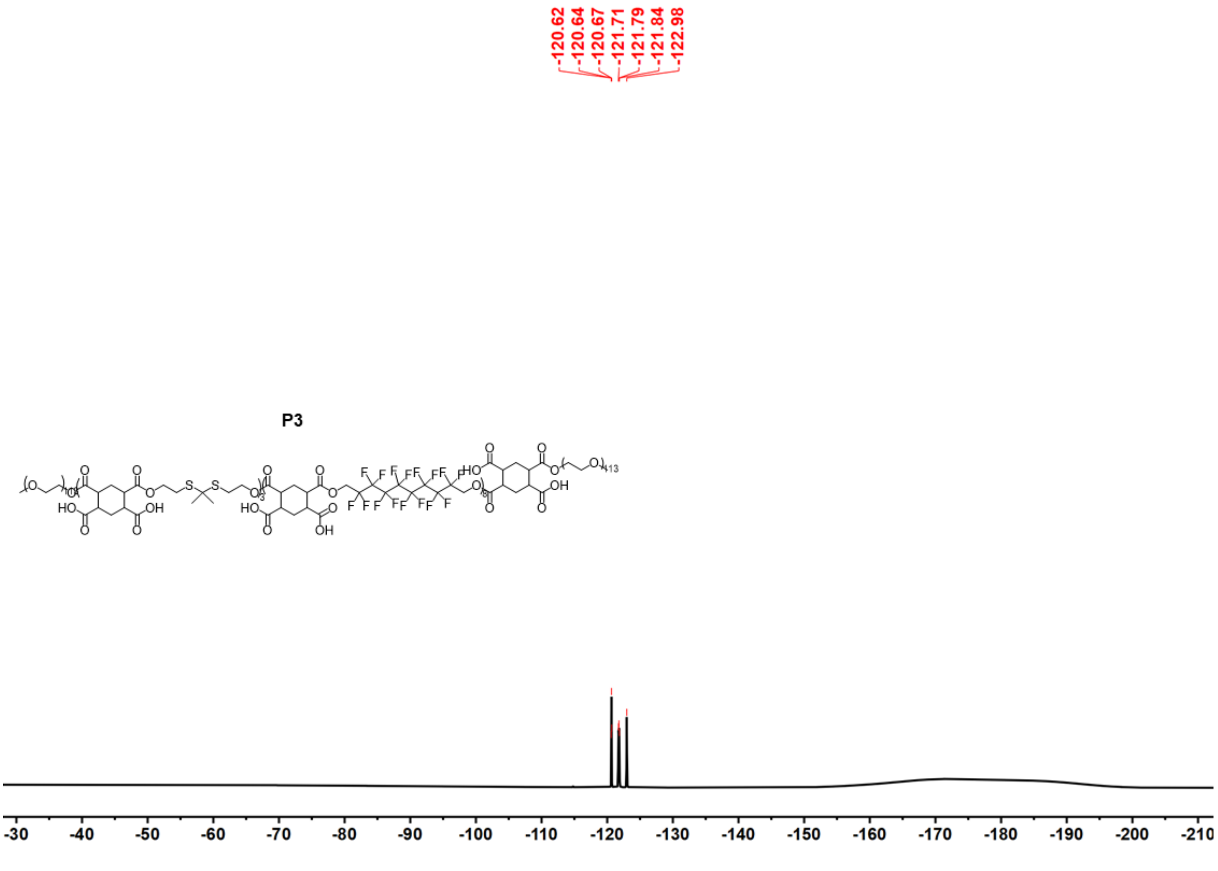

Supplementary Figure 19. ^{1}H-NMR and ^{19}F NMR spectrums of P3 in DMSO-d_6.

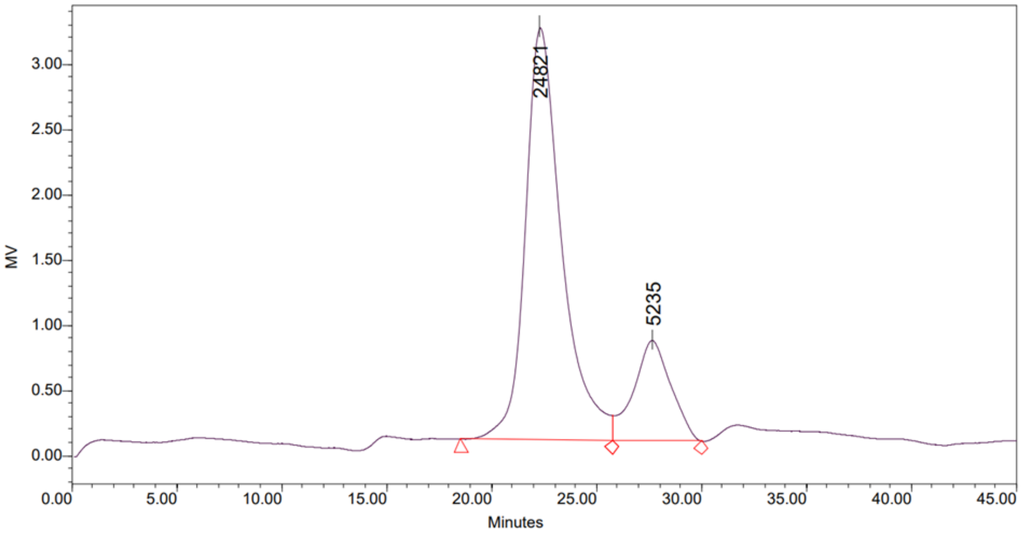

Supplementary Figure 20. GPC of P3 in DMF.

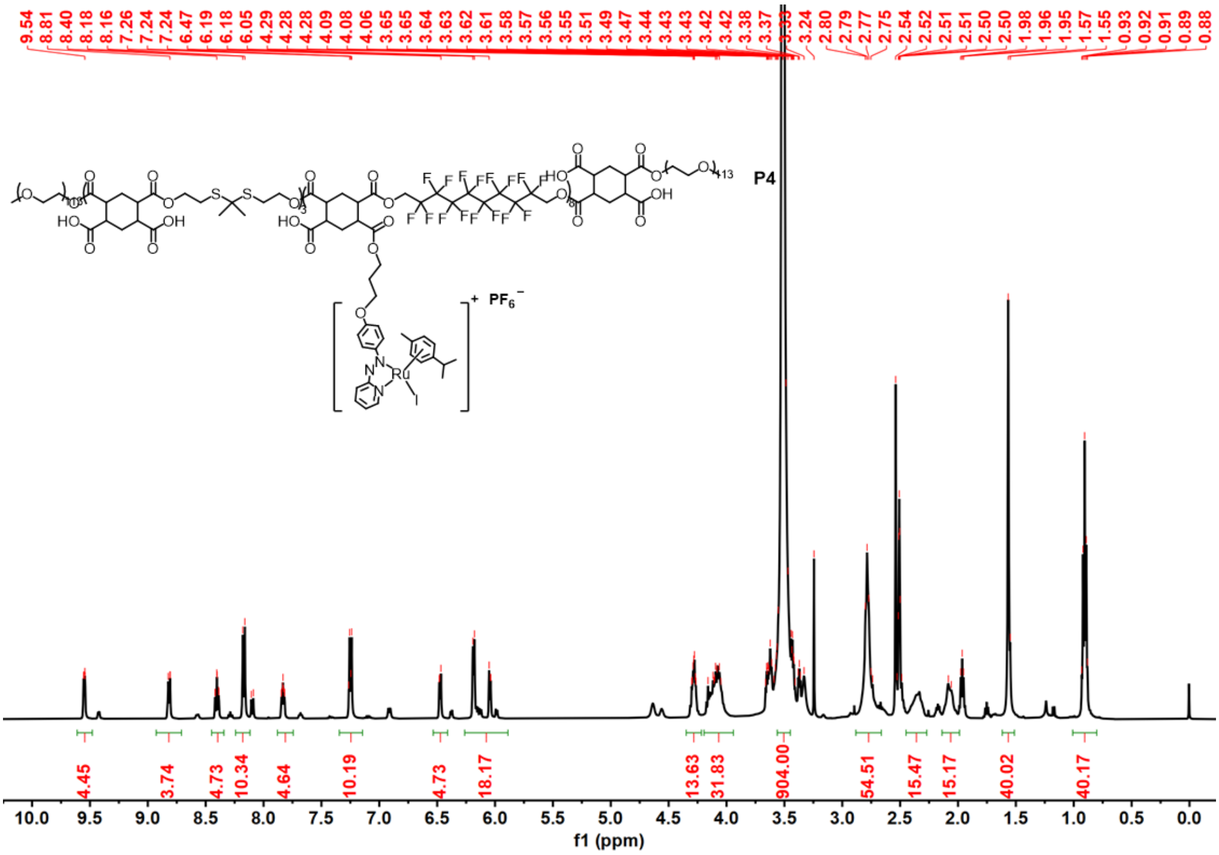

Supplementary Figure 21. ^{1}H-NMR spectrum of P4 in DMSO-d_6.

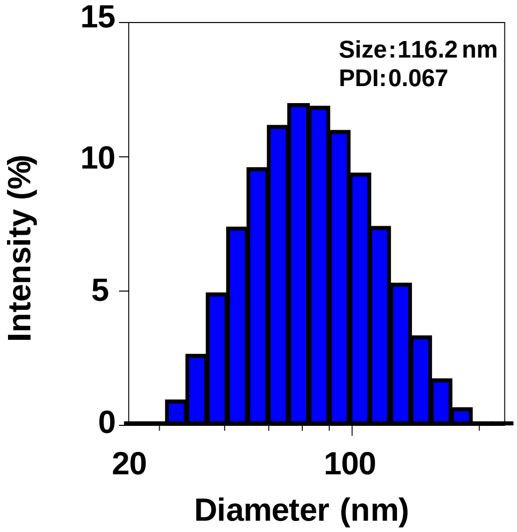

Supplementary Figure 22. Size distribution of NP2 detected by dynamic light scattering (DLS) (n = 3 independent experiments).

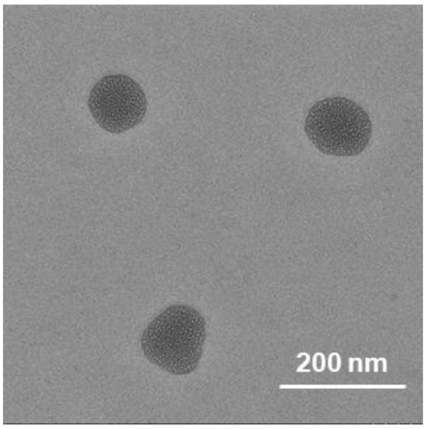

Supplementary Figure 23. Representative transmission electron microscope (TEM) image of NP2.

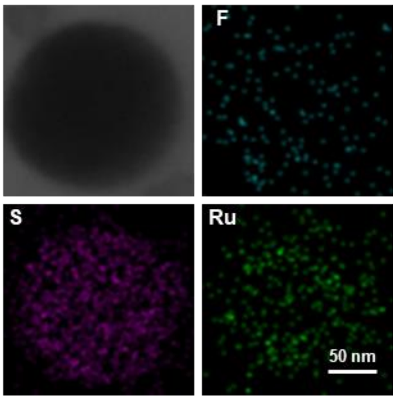

Supplementary Figure 24. Elemental distribution of NP2 analyzed by energy-dispersive X-ray spectroscopy.

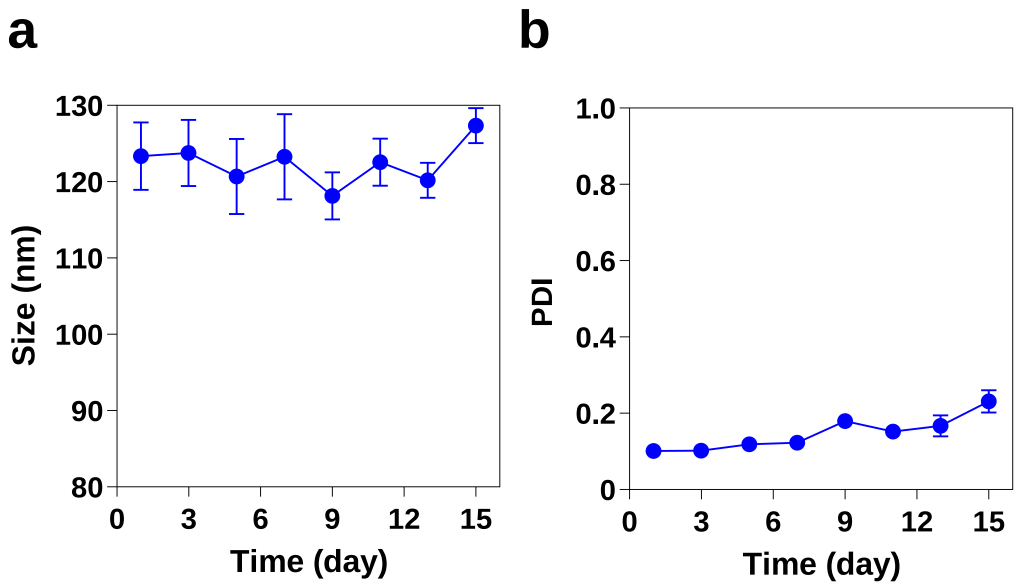

Supplementary Figure 25. Stability assessment of NP4 by DLS measurements. a) Change in average particle size of NP4 over 14 days. b) Change in polydispersity index (PDI) of NP4 over 14 days (n = 3 independent experiments).

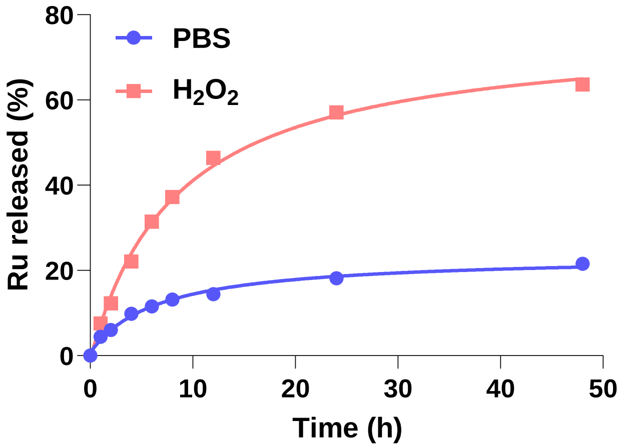

Supplementary Figure 26. Representative ruthenium (Ru) release profile of NP4 upon incubation in PBS or hydrogen peroxide (H_2O_2, 10 mM) determined by Inductively coupled plasma-Mass Spectrometry (ICP-MS).

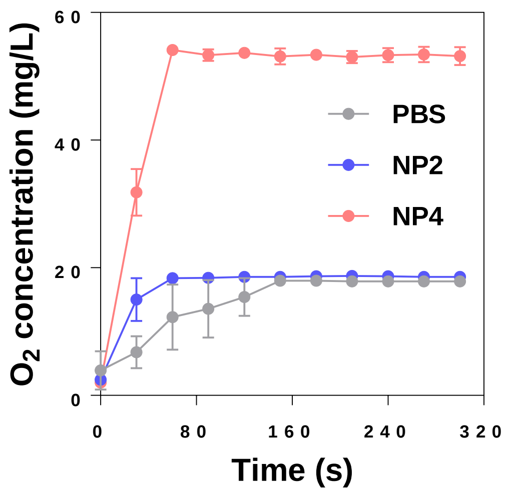

Supplementary Figure 27. Determination of oxygen storage capacity of the compounds (n = 3 independent experiments).

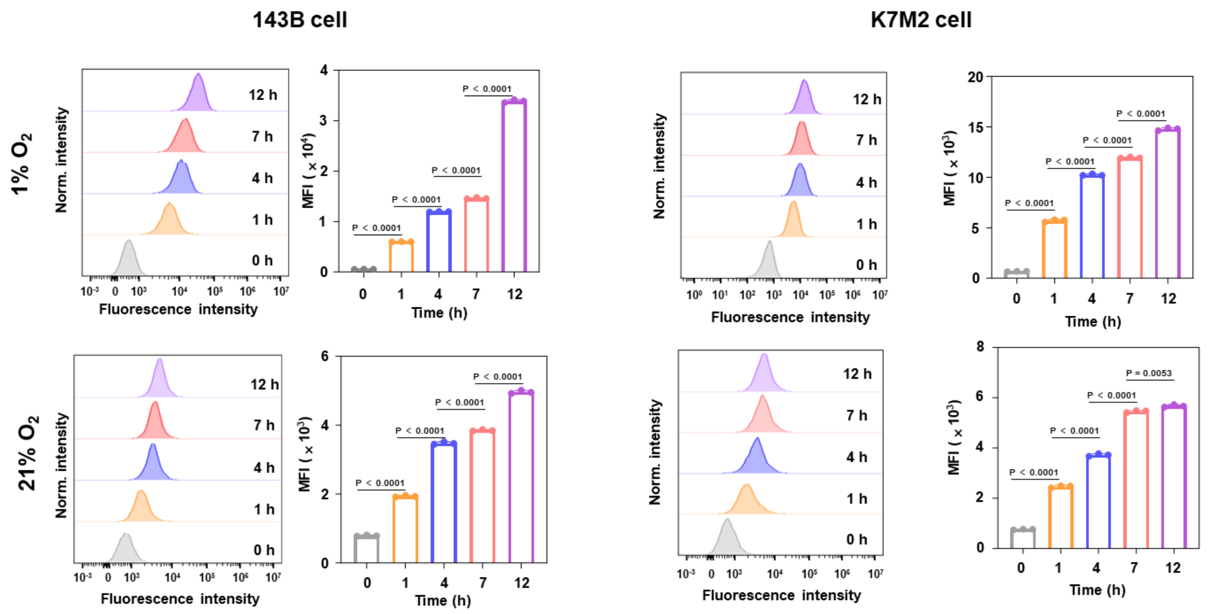

Supplementary Figure 28. The cellular uptake of nanoparticles was detected in K7M2 cells or 143B cells under 1% O_2 and 21% O_2 conditions by flow cytometry, n = 3 independent experiments, Data are presented as mean ± standard deviation (SD). Statistical significance between every two groups was calculated by T-test, the statistical test used was two-sided..

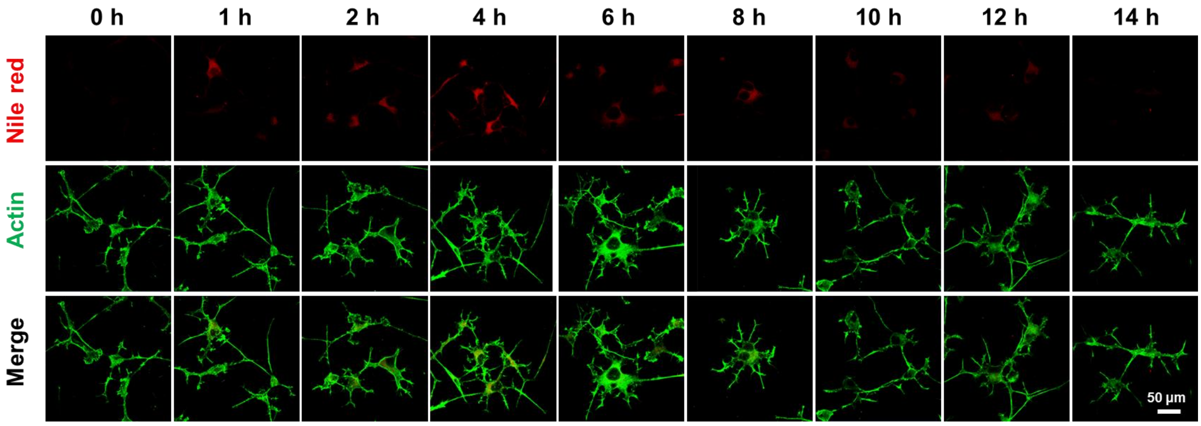

Supplementary Figure 29. Confocal laser scanning microscopy (CLSM) images of 143B cells incubated with NP4-NR for different times.

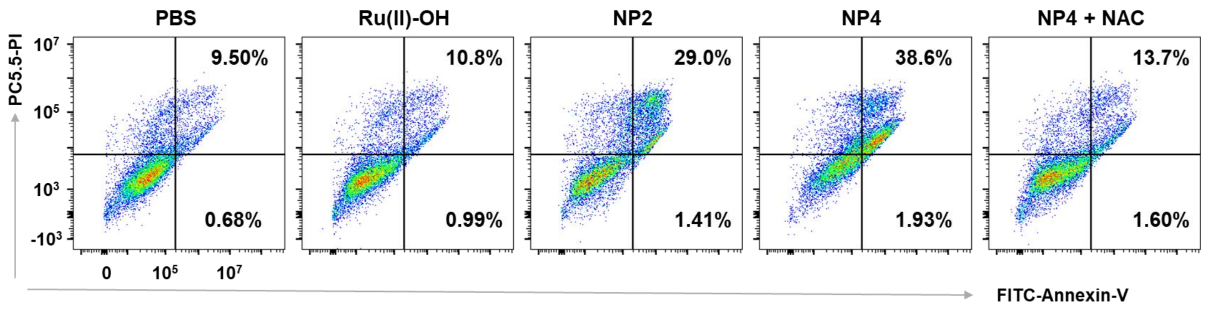

Supplementary Figure 30. Flow cytometry plots of 143B cells under hypoxic conditions treated with of Ru(II)-OH, NP2, NP4, and NP4 +NAC (10 μM Ru) for 24 h and stained with Annexin V-FITC and propidium iodide.

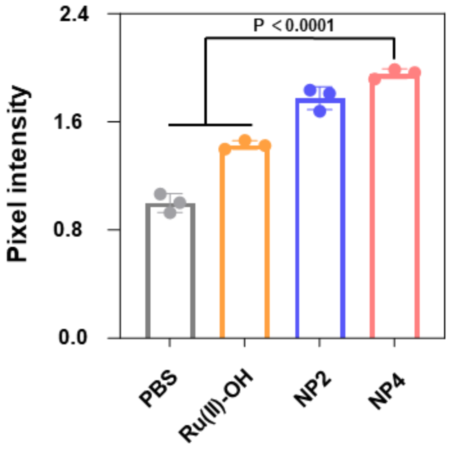

Supplementary Figure 31. Fluorescence semi-quantitative analysis of 143B cells under hypoxic conditions treated with of Ru(II)-OH, NP2, and NP4 (10 μM Ru) for 6 h and stained with the lipid peroxide specific probe C11-BODIPY (green) and DAPI (blue, nucleus), n = 3 independent experiments. Data are presented as mean ± standard deviation (SD). Statistical significance between every two groups was calculated by T-test, the statistical test used was two-sided.

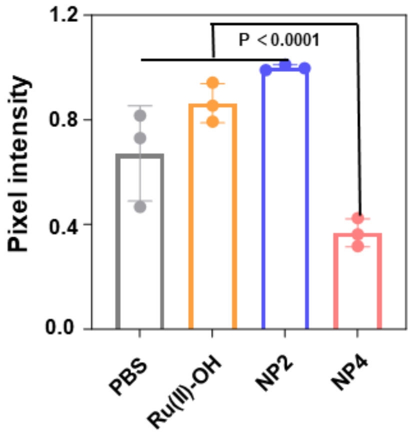

Supplementary Figure 32. Fluorescence semi-quantitative analysis of 143B cells under hypoxic conditions treated with of Ru(II)-OH, NP2, and NP4 (10 μM Ru) for 12 h and stained with the hypoxia probe pimonidazole hydrochloride (green, hypoxia areas) and DAPI (blue, nucleus), n = 3 independent experiments. Data are presented as mean ± standard deviation (SD). Statistical significance between every two groups was calculated by T-test, the statistical test used was two-sided.

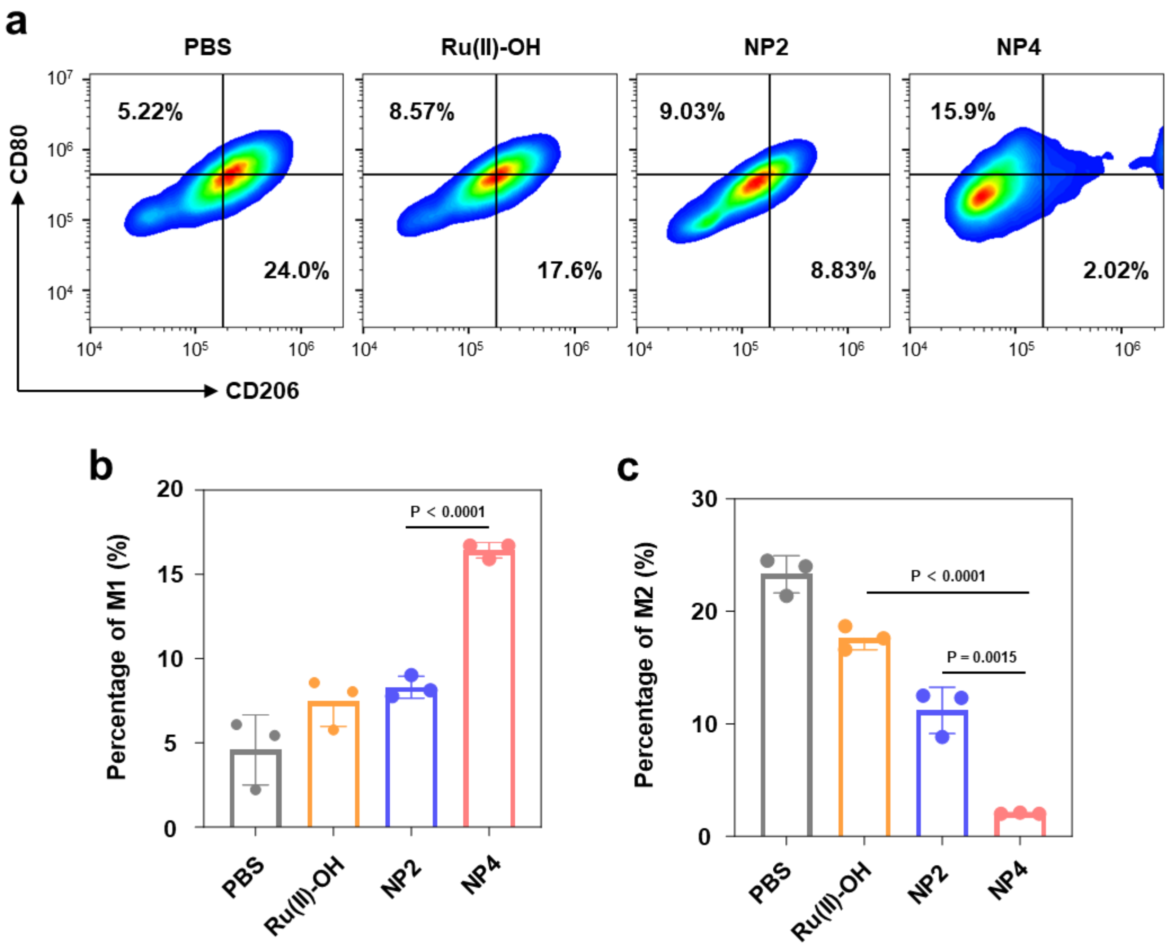

Supplementary Figure 33. The differentiation of tumor-associated macrophages (TAM). a) Flow cytometric analysis of $CD80^+$ $CD206^+$ bone marrow-derived macrophage (BMDM) gating on $CD11c^+$cells co-cultured with K7M2 cells with various pretreatments. b) Percentage of M1-macrophages ($CD11c^+$ $CD80^+$). c) Percentage of M2-macrophages ($CD11c^+$ $CD206^+$). n = 3 independent experiments. Data are presented as mean ± standard deviation (SD). Statistical significance between every two groups was calculated by T-test, the statistical test used was two-sided.

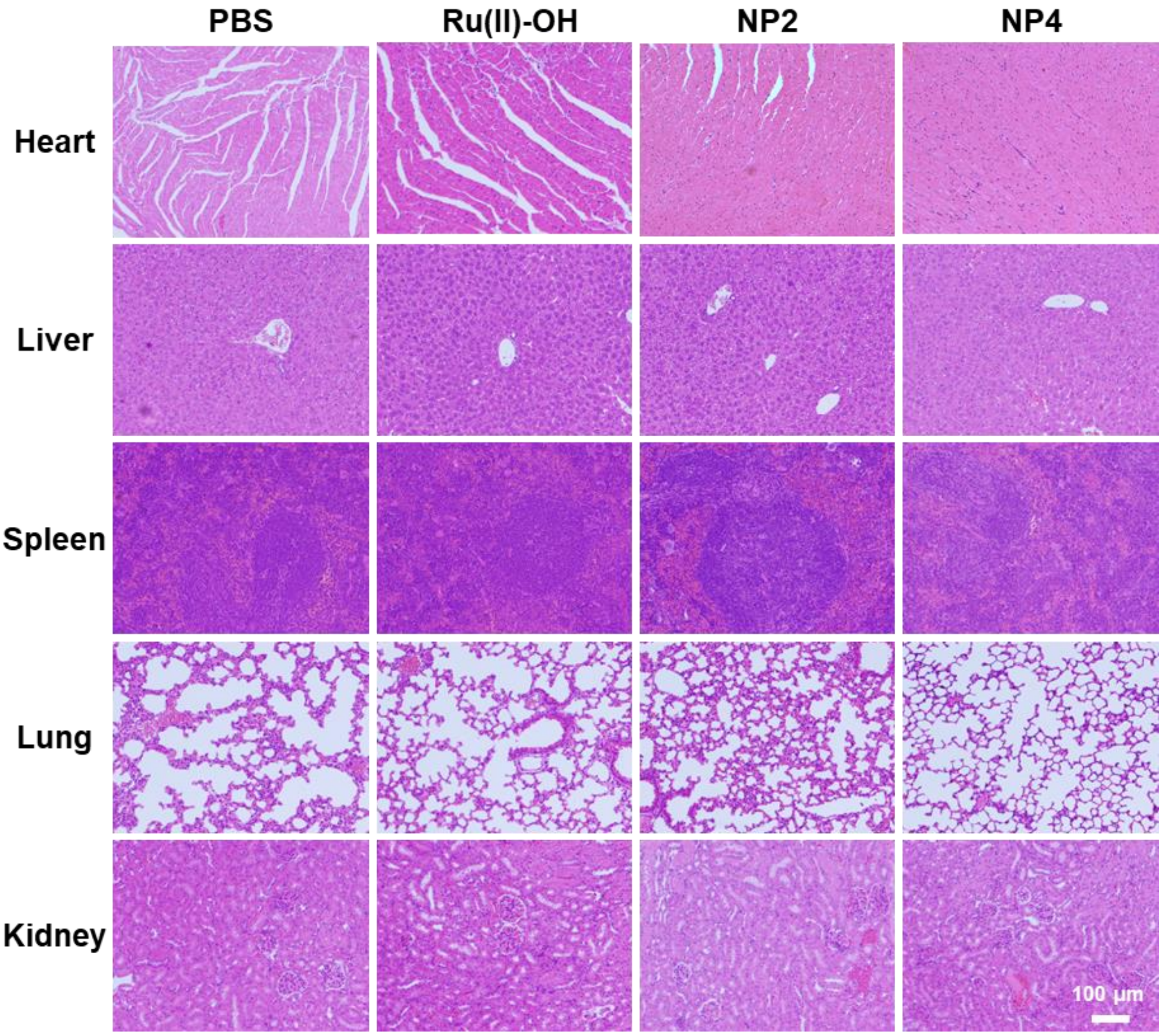

Supplementary Figure 34. Hematoxylin and eosin (H&E) stain of the major organs (heart, liver, spleen, lung, and kidney) of healthy mouse models upon treatment with PBS, Ru(II)-OH, NP2, and NP4 (2 mg/kg) on the days 1, 4, and 7. On day 14, the mice were sacrificed and the major organs were histologically analyzed (n = 3 mice).

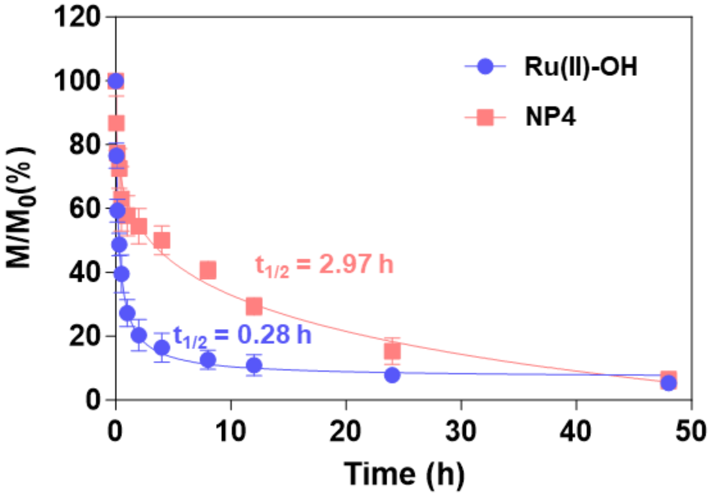

Supplementary Figure 35. Blood circulation half life time of Ru(II)-OH or NP4 determined by ICP-MS, (n = 3 mice).

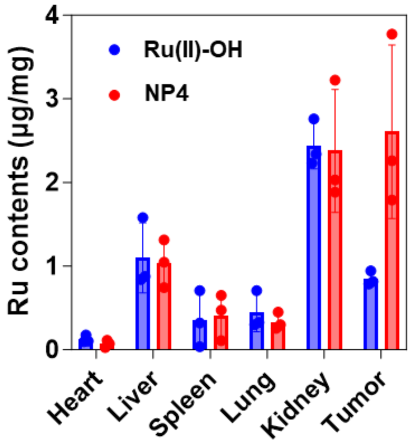

Supplementary Figure 36. Ruthenium contents in different organs and the tumor tissues of mice treated with NP4 and Ru(II)-OH determined by ICP-MS (n = 3 mice).

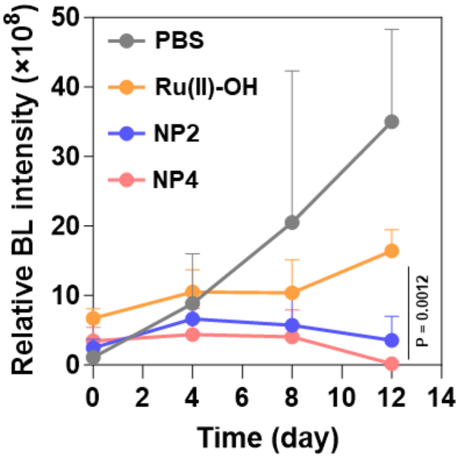

Supplementary Figure 37. Relative bioluminescent (BL) intensity upon various treatments in an orthotopic K7M2 osteosarcoma model, n = 3 mice. Data are presented as mean ± standard deviation (SD). Statistical significance between every two groups was calculated by T-test, the statistical test used was two-sided.

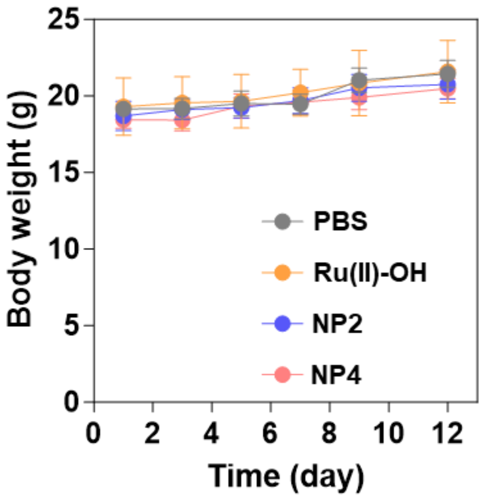

Supplementary Figure 38. Body weight upon various treatments in orthotopic K7M2 osteosarcoma model (n = 5 mice).

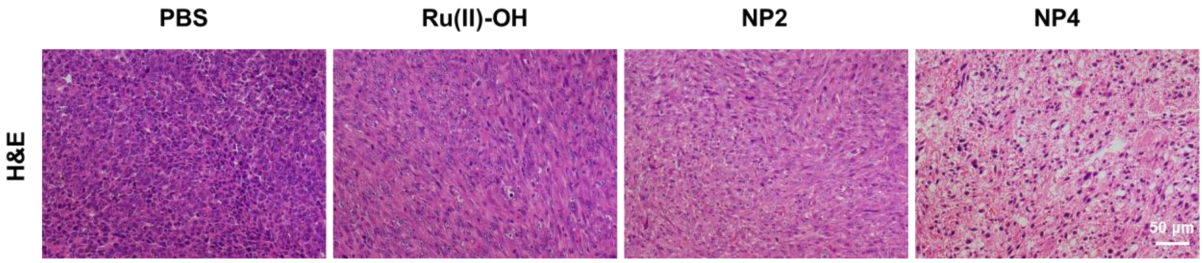

Supplementary Figure 39. H&E staining of tumor tissues after treatment in orthotopic K7M2 osteosarcoma model.

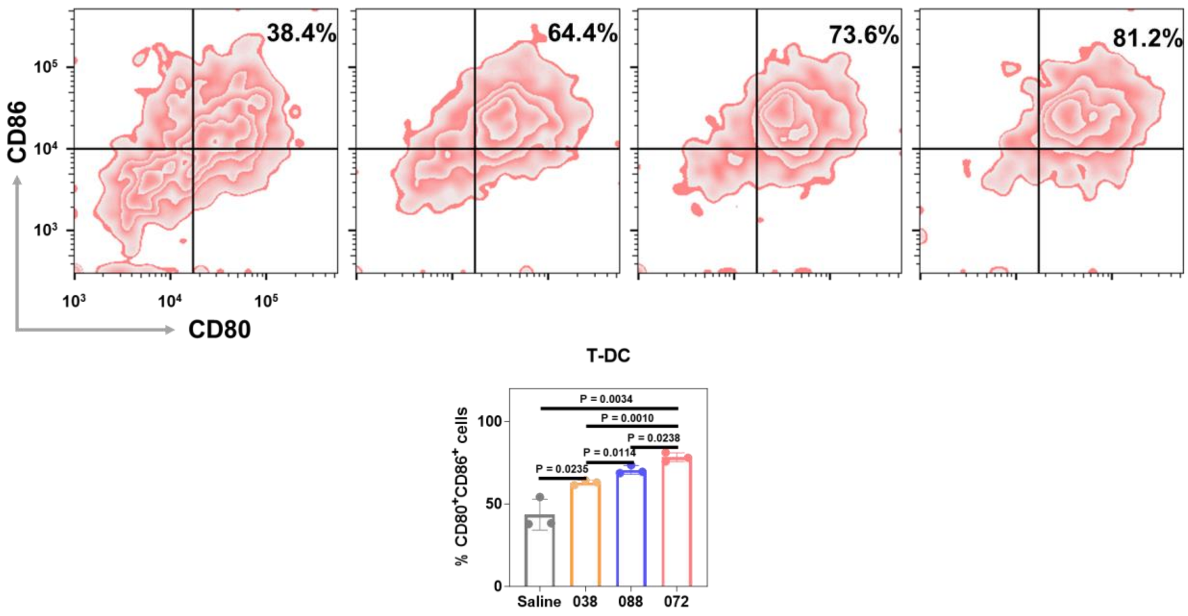

Supplementary Figure 40. Analysis of the levels of activated dendritic cells ($CD80^+$, $CD86^+$) in tumor obtained from variously treated K7M2 mouse models, n = 3 mice. Data are presented as mean ± standard deviation (SD). Statistical significance between every two groups was calculated by T-test, the statistical test used was two-sided.

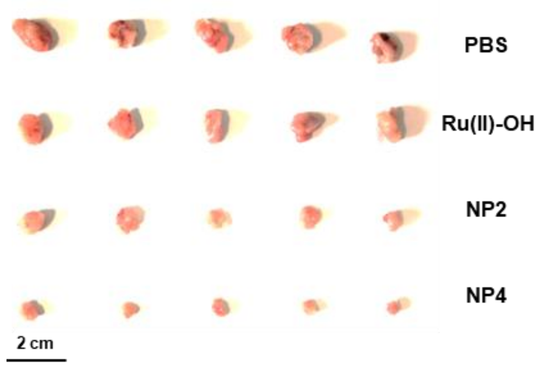

Supplementary Figure 41. Photographs of tumors obtained from treated 143B tumor-bearing mice.

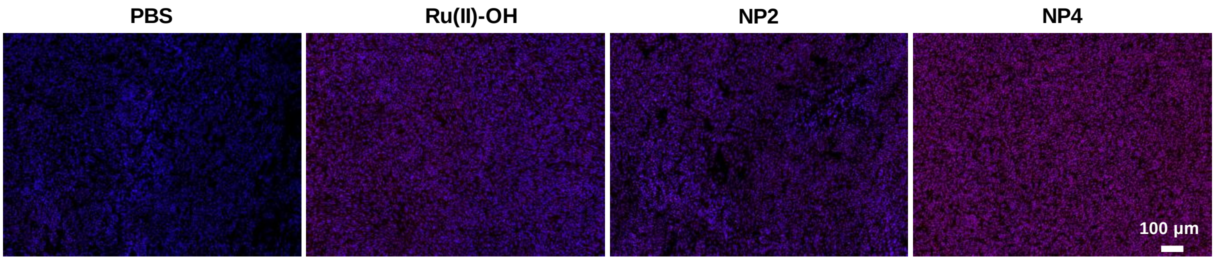

Supplementary Figure 42. Reactive oxygen species (ROS) (red) and DAPI (blue) staining of tumor tissues after treatment in a patient-derived osteosarcoma xenograft mouse model.

Supplementary Table 1. Parameters of the catalytic performance of Ru(II)-OH.

Catalyst	Temperature	Condition	Apparent k (h^{-1})	TON	TOF (h^{-1})
Ru(II)-OH	37 ℃	50 μM Ru	0.0257	38.2	0.3141

Supplementary Table 2. The IC_{50} of Ru(II)-OH, NP2, and NP4 on 143B and K7M2 cells under 21% O_2 and 1% O_2

IC_{50}(μM)	21% O_2		1% O_2	
Complex	143B	K7M2	143B	K7M2
Ru(II)-OH	7.57±0.59	10.43±0.76	> 15	> 15
NP2	6.88±0.44	8.30±0.17	> 15	> 15
NP4	6.06±0.42	7.96±0.43	6.57±0.08	2.09±0.59

Supplementary Table 3. Cartesian coordinates of the optimized structure of complex

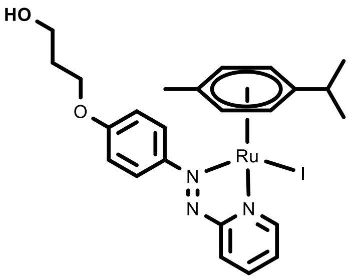

C 9.77000000 2.57700000 4.63000000

C 10.19500000 3.36200000 5.71900000

C 10.29200000 2.84000000 7.00800000

C 9.95900000 1.47100000 7.21000000

C 9.53800000 0.66800000 6.15400000

C 9.43000000 1.21700000 4.82200000

C 4.43300000 2.31300000 4.38400000

C 4.12200000 0.99000000 4.23700000

C 5.00000000 0.02700000 4.71100000

C 6.16900000 0.41800000 5.32400000

N 6.50400000 1.71900000 5.45600000

C 5.64200000 2.65400000 5.00000000

N 5.97100000 3.98600000 5.17000000

N 7.13200000 4.14700000 5.75400000

C 7.53500000 5.46600000 5.94000000

C 6.98300000 6.51800000 5.18100000

C 7.38400000 7.79100000 5.35000000

C 8.37100000 8.12700000 6.32900000

C 8.89900000 7.07500000 7.10500000

C 8.47400000 5.77300000 6.90600000

I 6.97200000 2.48800000 8.61500000

Ru 8.24000000 2.47300000 6.23400000

H 10.04210000 1.09590000 8.20860000

H 10.62190000 3.49220000 7.78950000

H 9.68420000 2.96690000 3.63730000

H 9.10280000 0.56740000 4.03730000

H 6.83090000 -0.33060000 5.70670000

H 4.77060000 -1.01230000 4.60110000

H 3.20900000 0.70000000 3.76030000

H 3.76510000 3.07260000 4.03490000

H 8.88010000 4.98970000 7.51140000

H 9.63410000 7.28480000 7.85370000

H 6.95850000 8.56300000 4.74340000

H 6.22850000 6.29920000 4.45450000

C 10.54400000 4.83100000 5.41600000

H 9.80310000 5.24680000 4.76560000

H 10.56930000 5.38730000 6.32970000

H 11.50220000 4.87960000 4.94230000

C 9.20160000 -0.79950000 6.47780000

H 8.45590000 -1.15310000 5.79680000

C 8.66840000 -0.89790000 7.91910000

H 7.71050000 -1.37450000 7.91340000

H 9.34890000 -1.47200000 8.51270000

H 8.57540000 0.08440000 8.33290000

C 10.47160000 -1.65970000 6.34030000

H 10.97320000 -1.41180000 5.42830000

H 11.12220000 -1.46980000 7.16830000

H 10.20150000 -2.69510000 6.32890000

O 8.80520000 9.47690000 6.51360000

C 10.23450000 9.52130000 6.51960000

H 10.60140000 9.07440000 7.41990000

H 10.61160000 8.98340000 5.67500000

C 10.70290000 10.98650000 6.44600000

H 10.28000000 11.53750000 7.25990000

H 10.38360000 11.41690000 5.51980000

O 12.12970000 11.03540000 6.52760000

H 12.41970000 11.95060000 6.52350000

Supplementary Table 4. Cartesian coordinates of the optimized structure of complex

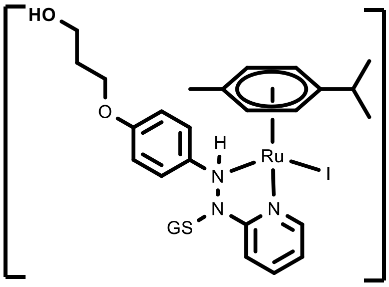

C 9.79860000 2.65040000 4.13830000

C 10.27580000 3.40480000 5.22730000

C 10.44860000 2.84300000 6.49130000

C 10.13990000 1.46470000 6.66670000

C 9.66860000 0.69120000 5.60980000

C 9.48220000 1.28130000 4.30430000

C 4.45880000 2.33580000 4.17710000

C 4.15230000 1.01490000 4.00450000

C 5.06400000 0.04690000 4.39800000

C 6.26140000 0.43090000 4.95830000

N 6.59120000 1.73060000 5.11400000

C 5.69680000 2.67020000 4.73660000

N 6.02240000 3.99960000 4.93150000

N 7.21230000 4.15460000 5.45580000

C 7.61280000 5.47130000 5.66220000

C 7.01010000 6.54090000 4.96930000

C 7.40810000 7.81220000 5.15730000

C 8.44450000 8.12760000 6.09110000

C 9.02420000 7.05720000 6.80230000

C 8.60090000 5.75760000 6.58470000

I 7.22580000 2.40340000 8.26580000

Ru 8.36050000 2.47840000 5.81960000

H 10.28150000 1.05880000 7.64650000

H 10.81520000 3.47350000 7.27430000

H 9.65450000 3.07090000 3.16500000

H 9.11820000 0.65360000 3.51810000

H 6.95030000 -0.32220000 5.27960000

H 4.83840000 -0.99080000 4.26710000

H 3.21710000 0.73020000 3.56940000

H 3.76570000 3.09880000 3.89010000

H 9.04700000 4.95990000 7.14110000

H 9.79750000 7.25100000 7.51590000

H 6.94270000 8.59850000 4.60040000

H 6.21870000 6.33720000 4.27850000

C 10.59400000 4.88650000 4.95350000

H 9.81450000 5.31470000 4.35860000

H 10.66460000 5.41360000 5.88200000

H 11.52410000 4.96070000 4.42970000

C 9.36420000 -0.78950000 5.90370000

H 8.58530000 -1.12930000 5.25360000

C 8.91230000 -0.93970000 7.36820000

H 7.95990000 -1.42640000 7.39960000

H 9.62980000 -1.52500000 7.90450000

H 8.83320000 0.02780000 7.81820000

C 10.63250000 -1.63080000 5.66880000

H 11.08070000 -1.34840000 4.73920000

H 11.32600000 -1.46040000 6.46560000

H 10.37170000 -2.66820000 5.63860000

O 8.87580000 9.47560000 6.29530000

C 10.30280000 9.53540000 6.22430000

H 10.72290000 9.06410000 7.08810000

H 10.63760000 9.02900000 5.34330000

C 10.75290000 11.00730000 6.17280000

H 10.37050000 11.52730000 7.02620000

H 10.37900000 11.46360000 5.28000000

O 12.18150000 11.06920000 6.17750000

H 12.46240000 11.98720000 6.18730000

H 6.84710000 4.10850000 6.38560000

C 3.71920000 5.09570000 3.97240000

H 3.77850000 4.76930000 2.95500000

H 3.09820000 4.42360000 4.52690000

S 5.13070000 5.11660000 4.58780000

C 3.11610000 6.51180000 4.02280000

N 3.74690000 7.29720000 5.18770000

C 3.47160000 6.60210000 6.45330000

H 4.26380000 8.14850000 5.09710000

O 2.71760000 5.38700000 6.45240000

C 3.99520000 7.18230000 7.78030000

H 5.06280000 7.24380000 7.74600000

H 3.58620000 8.15990000 7.92810000

C 3.57070000 6.26700000 8.94370000

H 2.50310000 6.20550000 8.97790000

H 3.97970000 5.28940000 8.79590000

C 4.09430000 6.84730000 10.27070000

H 3.68530000 7.82490000 10.41850000

N 5.56110000 6.93180000 10.22370000

H 5.84820000 7.86540000 10.43810000

H 5.95300000 6.30050000 10.89290000

H 5.87990000 6.68710000 9.30800000

C 3.66990000 5.93200000 11.43410000

O 2.90560000 4.75190000 11.17340000

O 4.04010000 6.26220000 12.77540000

H 2.05840000 6.44400000 4.16980000

C 3.40450000 7.24020000 2.69690000

O 4.15850000 8.45530000 2.69790000

N 2.90480000 6.68630000 1.43030000

H 3.28700000 5.77260000 1.29220000

C 3.30990000 7.56000000 0.31970000

H 2.90090000 8.53760000 0.46750000

H 4.37760000 7.62150000 0.28550000

C 2.78640000 6.97970000 -1.00720000

O 3.05420000 7.65600000 -2.23840000

O 2.03240000 5.76470000 -1.00820000

Supplementary Table 5. Cartesian coordinates of the optimized structure of complex

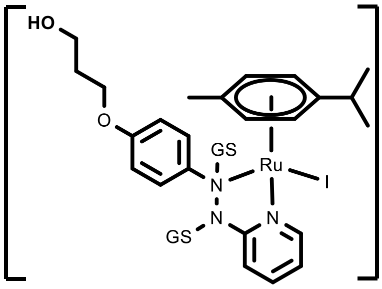

C 9.79860000 2.65040000 4.13830000

C 10.27580000 3.40480000 5.22730000

C 10.44860000 2.84300000 6.49130000

C 10.13990000 1.46470000 6.66670000

C 9.66860000 0.69120000 5.60980000

C 9.48220000 1.28130000 4.30430000

C 4.45880000 2.33580000 4.17710000

C 4.15230000 1.01490000 4.00450000

C 5.06400000 0.04690000 4.39800000

C 6.26140000 0.43090000 4.95830000

N 6.59120000 1.73060000 5.11400000

C 5.69680000 2.67020000 4.73660000

N 6.02240000 3.99960000 4.93150000

N 7.21230000 4.15460000 5.45580000

C 7.61280000 5.47130000 5.66220000

C 7.01010000 6.54090000 4.96930000

C 7.40810000 7.81220000 5.15730000

C 8.44450000 8.12760000 6.09110000

C 9.02420000 7.05720000 6.80230000

C 8.60090000 5.75760000 6.58470000

I 7.22580000 2.40340000 8.26580000

Ru 8.36050000 2.47840000 5.81960000

H 10.28150000 1.05880000 7.64650000

H 10.81520000 3.47350000 7.27430000

H 9.65450000 3.07090000 3.16500000

H 9.11820000 0.65360000 3.51810000

H 6.95030000 -0.32220000 5.27960000

H 4.83840000 -0.99080000 4.26710000

H 3.21710000 0.73020000 3.56940000

H 3.76570000 3.09880000 3.89010000

H 9.04700000 4.95990000 7.14110000

H 9.79750000 7.25100000 7.51590000

H 6.94270000 8.59850000 4.60040000

H 6.21870000 6.33720000 4.27850000

C 10.59400000 4.88650000 4.95350000

H 9.81450000 5.31470000 4.35860000

H 10.66460000 5.41360000 5.88200000

H 11.52410000 4.96070000 4.42970000

C 9.36420000 -0.78950000 5.90370000

H 8.58530000 -1.12930000 5.25360000

C 8.91230000 -0.93970000 7.36820000

H 7.95990000 -1.42640000 7.39960000

H 9.62980000 -1.52500000 7.90450000

H 8.83320000 0.02780000 7.81820000

C 10.63250000 -1.63080000 5.66880000

H 11.08070000 -1.34840000 4.73920000

H 11.32600000 -1.46040000 6.46560000

H 10.37170000 -2.66820000 5.63860000

O 8.87580000 9.47560000 6.29530000

C 10.30280000 9.53540000 6.22430000

H 10.72290000 9.06410000 7.08810000

H 10.63760000 9.02900000 5.34330000

C 10.75290000 11.00730000 6.17280000

H 10.37050000 11.52730000 7.02620000

H 10.37900000 11.46360000 5.28000000

O 12.18150000 11.06920000 6.17750000

H 12.46240000 11.98720000 6.18730000

C 7.69560000 4.12620000 3.36870000

H 8.16790000 4.91950000 3.90980000

H 8.00490000 3.18520000 3.77310000

S 6.16640000 4.26030000 3.49200000

C 8.09990000 4.20510000 1.88480000

N 6.92600000 3.73280000 1.00710000
C 6.58610000 2.34420000 1.34910000
H 6.46610000 4.28530000 0.31190000
O 7.32090000 1.65790000 2.36600000
C 5.43870000 1.62840000 0.61240000
H 4.52570000 2.16400000 0.76850000
H 5.65570000 1.59090000 -0.43470000
C 5.29320000 0.19570000 1.15800000
H 6.20630000 -0.33990000 1.00190000
H 5.07620000 0.23310000 2.20500000
C 4.14580000 -0.52010000 0.42120000
H 4.36280000 -0.55760000 -0.62590000
N 2.89140000 0.21560000 0.63560000
H 2.47480000 0.42880000 -0.24820000
H 2.26310000 -0.34630000 1.17350000
H 3.08310000 1.06510000 1.12720000
C 4.00030000 -1.95280000 0.96680000
O 4.86990000 -2.41490000 2.00360000
O 2.99540000 -2.82120000 0.43660000
H 8.94910000 3.57730000 1.71270000
C 8.45600000 5.66000000 1.52640000
O 7.72120000 6.34620000 0.50940000
N 9.55110000 6.34320000 2.22970000
H 9.34830000 6.37810000 3.20830000
C 9.69010000 7.71080000 1.70880000
H 9.90710000 7.67340000 0.66180000
H 8.77700000 8.24640000 1.86490000
C 10.83740000 8.42660000 2.44570000
O 11.16800000 9.77740000 2.11290000
O 11.57220000 7.74040000 3.46250000
S 6.25150000 4.65560000 6.77870000
C 6.11890000 5.71950000 7.88420000
H 7.06190000 6.20460000 8.02730000
H 5.38550000 6.44340000 7.59640000

C 5.68490000 5.04340000 9.19800000
N 4.90870000 3.75210000 8.87870000
H 5.05650000 5.70960000 9.75140000
C 6.93130000 4.69950000 10.03460000
C 3.71890000 4.08020000 8.08030000
H 5.17310000 2.83260000 9.16970000
O 7.20240000 3.34070000 10.38850000
N 7.84240000 5.76800000 10.46920000
O 3.44780000 5.43900000 7.72640000
C 2.76440000 2.96080000 7.62480000
H 8.21160000 6.23790000 9.66740000
C 8.94550000 5.18530000 11.24690000
H 3.29920000 2.26170000 7.01660000
H 2.36930000 2.45810000 8.48270000
C 1.60890000 3.57140000 6.81030000
H 8.55040000 4.68250000 12.10470000
H 9.48020000 4.48610000 10.63850000
C 9.90000000 6.30470000 11.70220000
H 1.07420000 4.27050000 7.41850000
H 2.00400000 4.07410000 5.95240000
C 0.65430000 2.45190000 6.35490000
O 11.05740000 5.98540000 12.47900000
O 9.62890000 7.66340000 11.34830000
H 0.25920000 1.94910000 7.21280000
N 1.38900000 1.49130000 5.51920000
C -0.50110000 3.06230000 5.54020000
H 1.27080000 0.56950000 5.88850000
H 1.03530000 1.52380000 4.58440000
H 2.36080000 1.72700000 5.51630000
O -0.55210000 4.47410000 5.31880000
O -1.52330000 2.21740000 5.00520000

Supplementary Table S6. Cartesian coordinates of the optimized structure of complex.

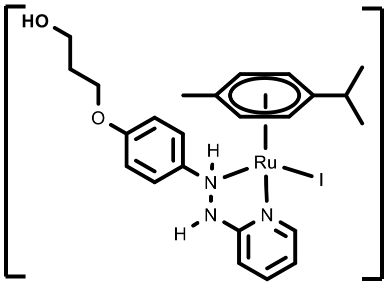

C 9.79860000 2.65040000 4.13830000

C 10.27580000 3.40480000 5.22730000

C 10.44860000 2.84300000 6.49130000

C 10.13990000 1.46470000 6.66670000

C 9.66860000 0.69120000 5.60980000

C 9.48220000 1.28130000 4.30430000

C 4.45880000 2.33580000 4.17710000

C 4.15230000 1.01490000 4.00450000

C 5.06400000 0.04690000 4.39800000

C 6.26140000 0.43090000 4.95830000

N 6.59120000 1.73060000 5.11400000

C 5.69680000 2.67020000 4.73660000

N 6.02240000 3.99960000 4.93150000

N 7.21230000 4.15460000 5.45580000

C 7.61280000 5.47130000 5.66220000

C 7.01010000 6.54090000 4.96930000

C 7.40810000 7.81220000 5.15730000

C 8.44450000 8.12760000 6.09110000

C 9.02420000 7.05720000 6.80230000

C 8.60090000 5.75760000 6.58470000

I 7.22580000 2.40340000 8.26580000

Ru 8.36050000 2.47840000 5.81960000

H 10.28150000 1.05880000 7.64650000

H 10.81520000 3.47350000 7.27430000

H 9.65450000 3.07090000 3.16500000

H 9.11820000 0.65360000 3.51810000

H 6.95030000 -0.32220000 5.27960000

H 4.83840000 -0.99080000 4.26710000

H 3.21710000 0.73020000 3.56940000

H 3.76570000 3.09880000 3.89010000

H 9.04700000 4.95990000 7.14110000

H 9.79750000 7.25100000 7.51590000

H 6.94270000 8.59850000 4.60040000

H 6.21870000 6.33720000 4.27850000

C 10.59400000 4.88650000 4.95350000

H 9.81450000 5.31470000 4.35860000

H 10.66460000 5.41360000 5.88200000

H 11.52410000 4.96070000 4.42970000

C 9.36420000 -0.78950000 5.90370000

H 8.58530000 -1.12930000 5.25360000

C 8.91230000 -0.93970000 7.36820000

H 7.95990000 -1.42640000 7.39960000

H 9.62980000 -1.52500000 7.90450000

H 8.83320000 0.02780000 7.81820000

C 10.63250000 -1.63080000 5.66880000

H 11.08070000 -1.34840000 4.73920000

H 11.32600000 -1.46040000 6.46560000

H 10.37170000 -2.66820000 5.63860000

O 8.87580000 9.47560000 6.29530000

C 10.30280000 9.53540000 6.22430000

H 10.72290000 9.06410000 7.08810000

H 10.63760000 9.02900000 5.34330000

C 10.75290000 11.00730000 6.17280000

H 10.37050000 11.52730000 7.02620000

H 10.37900000 11.46360000 5.28000000

O 12.18150000 11.06920000 6.17750000

H 12.46240000 11.98720000 6.18730000

H 6.84710000 4.10850000 6.38560000

H 5.41580000 4.75950000 4.6977000
